# 4-Thioribose Analogues of Adenosine Diphosphate Ribose (ADPr) Peptides

**DOI:** 10.1021/acs.orglett.3c01554

**Published:** 2023-06-20

**Authors:** Jerre M. Madern, Jim Voorneveld, Johannes G. M. Rack, Hans A. V. Kistemaker, Ivan Ahel, Gijsbert A. van der Marel, Jeroen D. C. Codée, Dmitri V. Filippov

**Affiliations:** † Leiden Institute of Chemistry, Leiden University, Einsteinweg 55, 2333 CC Leiden, The Netherlands; ‡ Sir William Dunn School of Pathology, University of Oxford, South Parks Road, Oxford OX1 3RE, United Kingdom

## Abstract

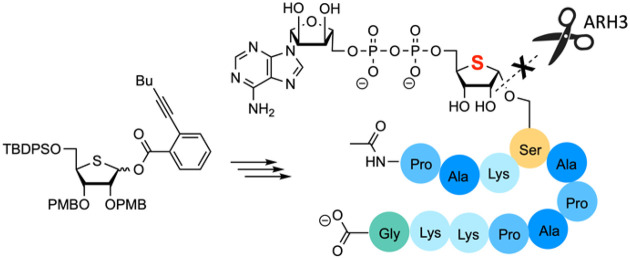

Adenosine diphosphate (ADP) ribosylation is an important post-translational modification (PTM) that plays a role in a wide variety of cellular processes. To study the enzymes responsible for the establishment, recognition, and removal of this PTM, stable analogues are invaluable tools. We describe the design and synthesis of a 4-thioribosyl APRr peptide that has been assembled by solid phase synthesis. The key 4-thioribosyl serine building block was obtained in a stereoselective glycosylation reaction using an alkynylbenzoate 4-thioribosyl donor.

Adenosine diphosphate (ADP) ribosylation is an important post-translational modification (PTM) that plays a role in a wide variety of cellular processes such as DNA repair, mitosis, apoptosis, transcription and metabolism, cellular stress, and immune response.^1^ This PTM is introduced on target proteins by (ADP-ribosyl)transferases (ARTs),^2^ and among the ARTs, PARPs make up the largest enzyme family^3^ and are responsible for adding either a single adenosine diphosphate-ribose (ADPr) unit or a poly-ADPr chain to the side chain of a specific amino acid. Although initially glutamate and aspartate were regarded as the prime acceptors for ADPr,^4^ recent studies have revealed serine to be the most common amino acid acceptor for this PTM (Figure 1).^5^ Arginine, lysine, cysteine, histidine, and tyrosine have also been shown to be ADP ribosylated.^6^

**Figure 1:**
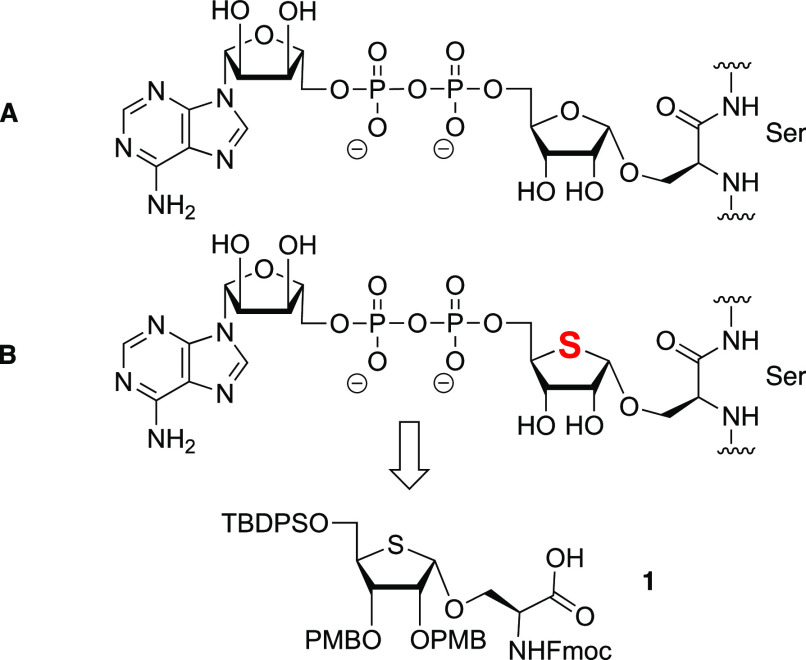
(A) ADPr serine. (B) 4-Thio-ADP-ribosylated serine and building block **1** for SPPS of 4-thio-ADPr serine peptides.

ADP ribosylation is a reversible PTM, and ADPr chains can be degraded by a poly-ADP-ribose glycohydrolase (PARG) down to the mono-ADPr protein.^7^ Cleavage of the mono-ADPr modification occurs under the action of (ADP-ribosyl)hydrolases that are specific for the amino acid to which the ADPr moiety is attached. For example, ARH3 has been shown to be responsible for the removal of ADPr form serine residues.^8^

Although the vital role of ADP ribosylation in health and disease has been recognized, our knowledge of ADP ribosylation lags far behind our understanding of other PTMs. The dynamic character and the chemical lability of the ADPr modifications contribute to our lack of knowledge regarding the molecular details underlying the processes regulated by ADP ribosylation. Sufficient quantities of structurally well-defined ADP-ribosylated peptide fragments that would allow the study of the ADPr biosynthesis machinery at the molecular level are difficult to isolate from natural sources. Organic synthesis has been used to procure ADPr fragments, as well as analogues and mimics equipped with a tag or fluorescent label, for biological testing.^9^ The glycosidic linkage in serine ADPr fragments represents a labile functionality that is potentially vulnerable to cleavage or anomerization reactions.^10^ We reasoned that close mimetics, in which the ribosyl serine bond is stabilized, can present valuable tool compounds for both functional and structural studies.

Thio-sugars, carbohydrates in which the ring oxygen has been replaced by a sulfur atom, have been shown to closely mimic the parent sugars, while they are significantly more stable toward acidic or enzymatic turnover.^11^ 4-Thioribosyl moieties have been used to stabilize DNA and RNA nucleotides^12^ and have been used in antiviral compounds as well as antibiotics.^13^ 4-Thioribose nicotinamide diphosphate (thio-NAD^+^) has proven to be a stabilized, competent NAD^+^ mimic.^14^

We present here the design and synthesis of a serine ADPr peptide in which the ribosyl linkage is stabilized by the incorporation of a 4-deoxy-4-thioribose moiety instead of the natural riboside (Figure 1). To this end, we have generated a 4-thio ribosyl serine building block (**1**), carrying protecting groups that enable its incorporation in solid phase ADPr peptide synthesis.^9a,15,16^ This building block carries *p*-methoxybenzyl (PMB) ethers at the 4-thioribose, as these can be removed at the end of the synthesis during global acidic deprotection, and a 5-*O*-*tert*-butyldiphenyl silyl (TBDPS) ether, which can be removed on resin to install the adenosine diphosphate moiety. The serine amine group is masked with a 9-fluorenylcarboxyl (Fmoc) group to enable standard solid phase peptide synthesis chemistry (SPPS). The building block has been used to assemble a short 4-thio-ADPr peptide corresponding to a fragment of histone H2B that contains a serine characterized *in vivo* as the ADPr acceptor site and ARH3 substrate.

The synthesis of building block **1** is depicted in Scheme 1. Key to the effective assembly of this building block is the formation of the *cis*-ribosyl linkage, which proved to be very challenging.^16^ To ensure the stereoselective installation of this linkage and generate a building block compatible with SPPS, we installed nonparticipating PMB ethers at C-2 and C-3. The synthesis of **1** started from d-ribose, which was transformed into fully protected allyl ribofuranoside **2**, possessing the required protecting group pattern around the ribose ring. Transposition of the ring oxygen by a sulfur atom was accomplished following the strategy developed by Minakawa et al.^17,18^ Thus, the anomeric allyl in **2** was removed by isomerization of the terminal alkene to deliver the corresponding enol ether, which was hydrolyzed using aqueous iodine. The resulting lactol was reduced to give diol **3**. To retain the stereochemistry at C-4, a double-inversion reaction sequence was performed, in which the diol was first transformed into dimesylate **4**, which was subsequently used to generate dibromide **5**. Substitution of both bromides and concomitant ring closure were affected by treatment of **5** with Na_2_S at increased temperatures to deliver 1-deoxy-4-thioriboside **6**. Oxidation of C-1 was achieved by treatment of the thioether with *m*-CPBA in DCM at −40 °C to form the intermediate sulfoxide. When the sulfoxide was heated to 100 °C in acetic anhydride, a Pummerer rearrangement was effected that led in a completely regioselective manner to 1-acetyl 4-thioribosyl **7**, which was explored as a ribosylation agent as described below. We also transformed acetate **7** into the corresponding *N*-phenyl trifluoroacetimidate **9**(19) by saponification and treatment with *N*-phenyl trifluoroacetimidoyl chloride.

**Scheme 1:**
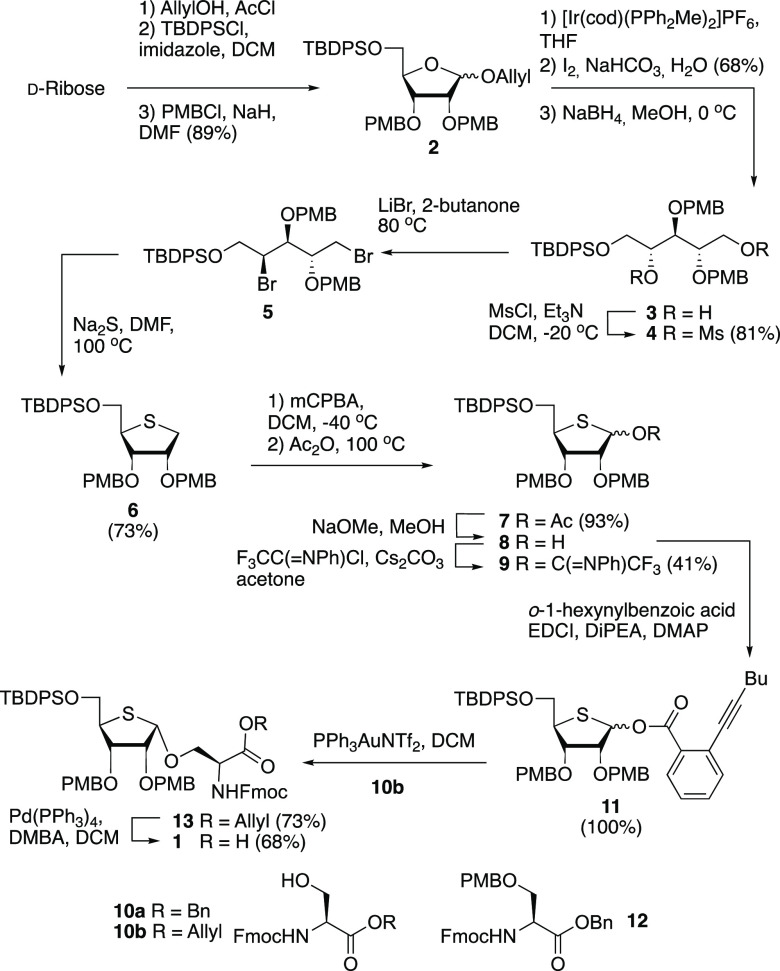
Synthesis of Key Building Block **1**

With the two donors in hand, we set out to forge the α-ribosyl linkage with serine acceptor **10a**. Unfortunately, under all (Lewis)-acidic conditions tested [TMSOTf/TBSOTf/HClO_4_-SiO_2_ (see the Supporting Information for full details)], we were not able to isolate the desired product in significant yield. Instead, the major product of these glycosylations turned out to be serine *p*-methoxybenzyl ether **12**, formed in ≤70% yield by PMB transfer from the donor. We therefore switched to a milder glycosylation methodology and generated alkynylbenzoate donor **11**.^20^ Hemithioacetal **9** was treated with EDCI and *o*-hexynylbenzoic acid in DCM to deliver donor **11** in quantitative yield. We selected allyl-protected acceptor **10b** for glycosylation because benzyl ester (as in **10a**) would be difficult to cleave by hydrogenolysis at the later stage of the synthesis due to potential poisoning of the Pd/C catalyst by the thioether of the thioribose. Gratifyingly, the alkynebenzoate donor and serine acceptor **10b** could be united under the aegis of a catalytic amount of PPh_3_AuNTf_2_ to deliver thioribosylated serine **13** in 73% yield with excellent *cis*-stereoselectivity (11:1 α:β). The allyl group was then removed using tetrakis(triphenylphosphine)palladium and 1,3-dimethylbarbituric acid (DMBA), followed by the addition of tetrahydrothiophene (THT) as a scavenger, liberating the carboxylic acid and yielding essential solid phase peptide synthesis building block **1** in 68% yield.

With the required building block **1** in hand, the solid phase assembly of mono-4-*S*-ADP-ribosylated peptide **19**, derived from the N-terminus of histone H2B, was undertaken.^10,21^ The standard Fmoc-based methodology was combined with amino acid building blocks carrying acid sensitive protecting groups on the side chains (Mtt for lysine and Trt for serine and threonine). As depicted in Scheme 2, the acid labile TentaGel S AC resin, preloaded with glycine, was elongated using the selected protected amino acid building blocks to generate full-length ribosylated immobilized peptide **14**. Next, the cleavage of the TBDPS protecting group was tested by using three different fluoride sources: TEA·3HF, HF·pyridine, and TBAF. Both TEA·3HF and HF·pyridine required reaction times of ≤16 h to fully remove the silyl protecting group, whereas a 1 M TBAF solution in THF ensured full deprotection in 30 min. The TBAF treatment was superior with regard to not only the reaction rate but also the quality of the product according to LC-MS analysis of the peptides after desilylation. The released primary hydroxyl of the ribose moiety was then phosphitylated with di(9-fluorenylmethyl)-*N*,*N*-diisopropylphosphoramidite (**15**) using 5-ethylthio-1*H*-tetrazole (ETT) as an activator to give the corresponding phosphite triester. Care must be exercised in the oxidation of the phosphite to the phosphotriester, as it has been observed in the synthesis of ADP-ribosylated peptides linked to a biotin tag, that the biotin sulfur be oxidized when using (1*S*)-(+)-(10-camphorsulfonyl)-oxaziridine (CSO).^9a^ Test reactions using protected thioribose substrates also showed quick and efficient oxidation of the thioether using CSO, whereas the use of tBuOOH led to minor or no oxidation of the thioether moiety. Gratifyingly, the oxidation of the immobilized phosphite using tBuOOH resulted in fully protected phosphoribosyl peptide **16**, as shown by LC-MS analysis after deprotection and cleavage from the solid support. On the basis of this favorable result, the Fm protecting groups in immobilized phosphotriester **16** were removed by treatment of the resin with 10% DBU in DMF. Monitoring the reaction progress by LC-MS showed that both Fm protecting groups were completely eliminated in 20 min to give the corresponding phosphomonoester. The assembly of the 4-thio-ADP-ribosylated peptide was continued with the installation of the pyrophosphate by coupling of the phosphomonoester with adenosine phosphoramidite **17** and the tBuOOH-mediated oxidation of the P^III^–P^V^ intermediate to give immobilized peptide **18**, containing a partially protected pyrophosphate moiety.^22^ The deprotection entailed the elimination of the cyanoethyl group from the pyrophosphate in **18** by treatment of the resin with 10% DBU in DMF, and cleavage of the silyl ethers with TBAF. Finally, the remaining protecting groups were removed with concomitant cleavage of the target 4-thio-ADP-ribosylated peptide from the resin by treatment with a 10% TFA solution in DCM containing 2.5% TIS as a scavenger for the trityl and *p*-methoxybenzyl carbocations. Monitoring of the deprotection by LC-MS analysis revealed that the Mtt and PMB protecting groups were split off instantly while the more stable Boc group on the exocyclic amine of adenosine needed at least 4 h to be removed. Purification with RP-HPLC of the obtained crude product led to the isolation of thio-MARylated peptide **19**, derived from N-terminal histone H2B in a 4.1% overall yield calculated from the resin loading.

**Scheme 2:**
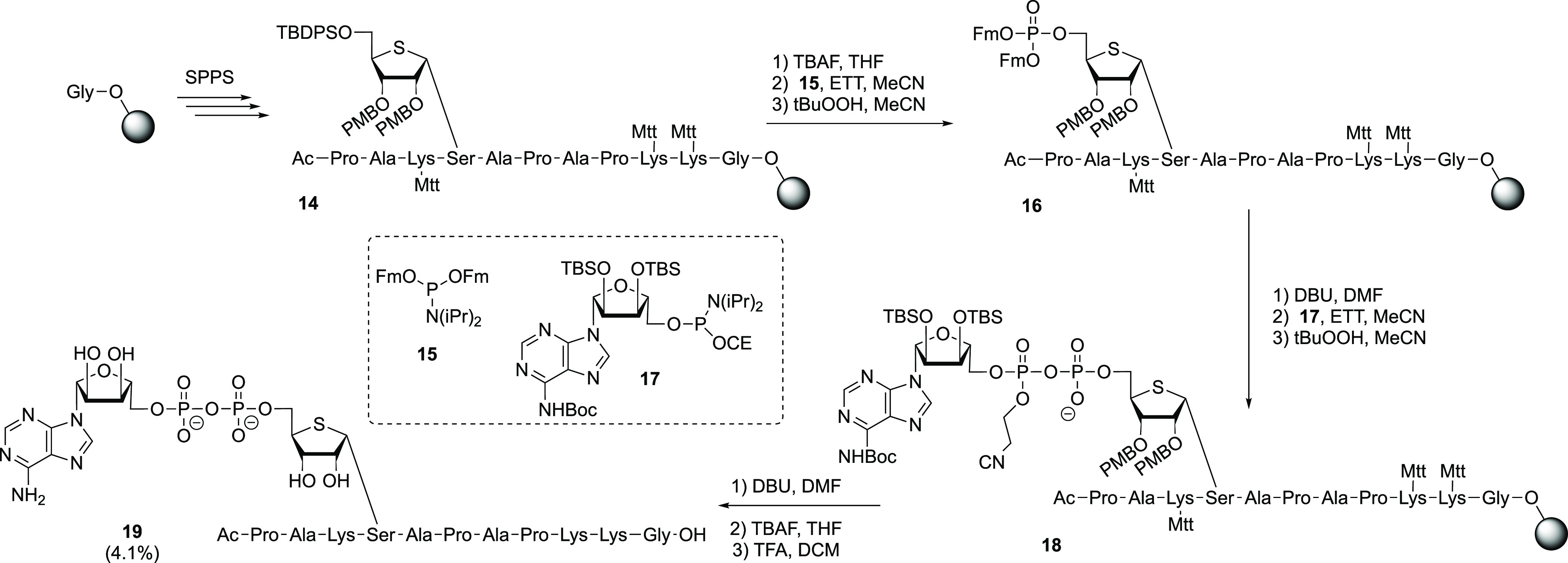
Solid Phase Peptide Synthesis of a 4-Thio-ADP-Ribosylated H2B Peptide Abbreviations: DBU, 1,8-diazabicyclo[5.4.0]undec-7-ene; ETT, 5-ethylthio-1*H*-tetrazole; Mtt, 4-methytrityl; SPPS, solid phase peptide synthesis; TBAF, tetra-*n*-butylammonium fluoride; TFA, trifluoroacetic acid.

Having synthesized Ser-thio-ADPr peptide **19**, we evaluated the Ser-ADPr isostere with respect to its sensitivity to enzymatic cleavage. ARH3 is known to hydrolyze the O-glycosidic linkage of Ser-ADPr and indeed could convert the Ser-ADPr control peptide efficiently (Figure 2). In contrast, our preliminary screen using a panel of human hydrolases utilizing an established luminescence reporter assay^10^ showed only marginal activity of ARH3 against Ser-thio-ADPr. While a complete hydrolysis of native Ser-ADPr was achieved in 45 min, only approximately 3% of the Ser-thio-ADPr counterpart was removed in the same amount of time. Note the ability of NudT16, which directly cleaves AMP from the modified peptide and acts as an internal control, is also severely weakened (to ∼9%) by the ribose to thioribose exchange. While we cannot fully exclude the possibility that the thioribose influences the enzyme–substrate interaction, our results suggest a strong stabilizing effect of the thio-sugar and the adjacent O-glycosidic bond.

**Figure 2:**
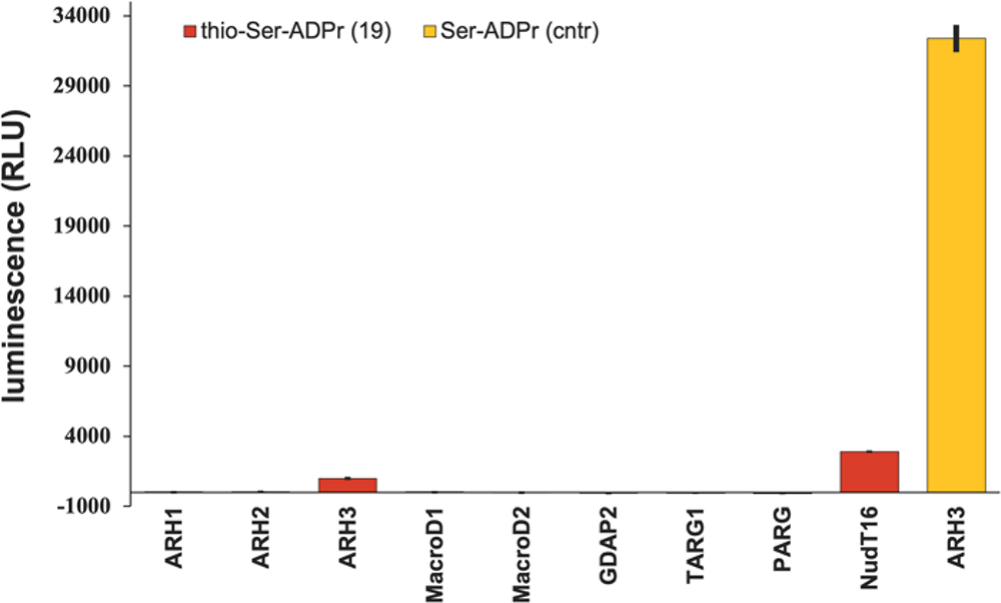
Enzymatic hydrolysis of the gycosidic linkage in thio-ADP-ribosylated peptide **19** and the *O*-ribose control. Enzymatic turnover of the peptide was assessed by measuring the AMP release directly (NudT16) or converting released thio-ADPr via NudT5 to AMP. AMP was measured using the AMP-Glo assay (Promega). Reactions were measured in triplicate ± the standard deviation.

In conclusion, we have designed and synthesized a close analogue of an ADP-ribosylated serine-containing peptide, in which the central ribosyl moiety has been replaced by a 4-thioribosyl unit. The incorporation of the sulfur atom renders the glycosidic linkages significantly more stable toward enzymatic degradation. The synthesis was accomplished through the glycosylation of a suitably protected serine using an alkynylbenzoate 4-thioribosyl donor, which could be activated under mild conditions, which did not jeopardize the acid labile PMB ethers, which were installed to guarantee the stereoselective formation of the *cis*-ribosyl linkage. The 4-thioribosylated serine building block could be used in the solid phase assembly of a model H2B peptide in which we constructed the central pyrophospate linkage using phosporamidite chemistry. After P(III)–P(IV) coupling, the oxidation of the phosphite intermediate could be effected using *t*-BuOOH as a mild oxidizing agent, to prevent oxidation of the thioether of the 4-thioribosyl moiety. The chemistry developed will allow for the incorporation of the stable mimic in various peptide sequences to generate probes for activity and structural biology studies on ARH3 and related hydrolases and to further illuminate the role of this fascinating protein PTM in health and disease.

## Data Availability

The data underlying this study are available in the published article and its Supporting Information.

## Supporting Information Available

Experimental procedures for all new compounds, optimization of serine glycosylation, solid phase peptide synthesis of **19**, and copies of NMR spectra (PDF)
