# Supplementary material for: 4-Thioribose Analogues of Adenosine Diphosphate Ribose (ADPr) Peptides
Source: Org Lett. 2023 Jun 20;25(27):4980–4. doi: 10.1021/acs.orglett.3c01554 (PMC10353035; doi:10.1021/acs.orglett.3c01554)

Supporting information

4-thioribose analogues of adenosine diphosphate ribose (ADPr) peptides

Jerre M. Madern[1], Jim Voorneveld[1], Johannes G. M. Rack[2], Ivan Ahel[2], Gijsbert A. van der Marel[1], Jeroen D. C. Codée[1], Dmitri V. Filippov[1]

[1]Leiden Institute of Chemistry, Leiden University, Einsteinweg 55, 2333 CC Leiden, The Netherlands

[2]Sir William Dunn School of Pathology, University of Oxford, South Parks Road, Oxford, OX1 3RE, United Kingdom

Table of contents

Explorative glycosylation reactions

Experimental

- General procedures
- Synthetic procedures
- Protein expression
- Hydrolase assay

NMR spectra

LCMS

Explorative glycosylation reactions

Table 1. Explorative 4-thioribosylation reactions.

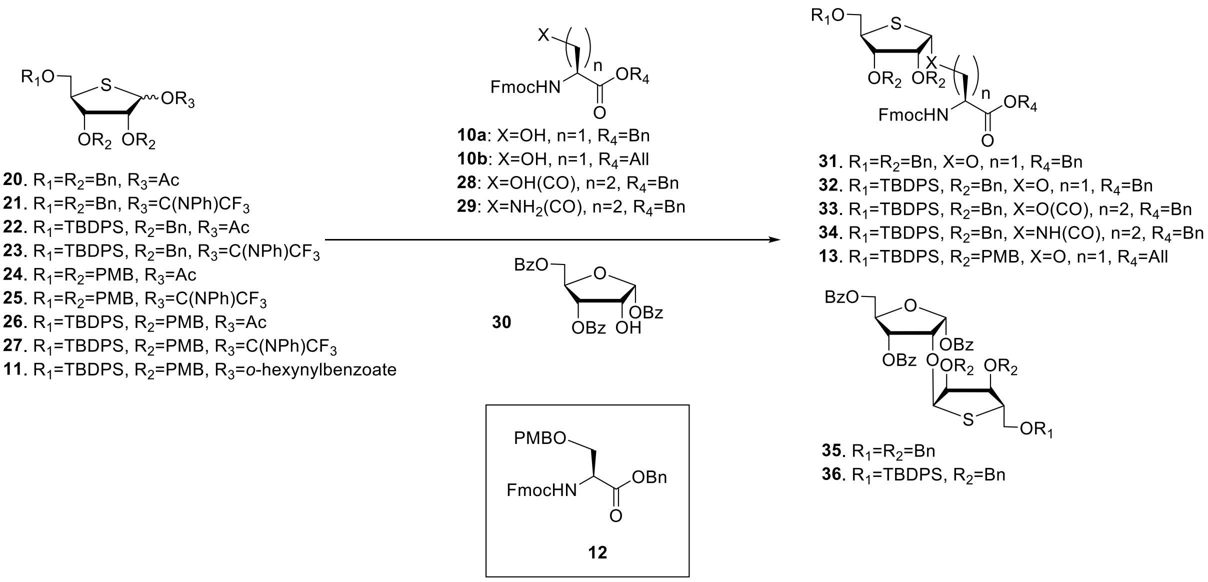

No.	*Donor*	*Acceptor*	*Product*	*Activator*	*Solvent*	*Temp (ºC)*	*RT (hr)*	*Yield (%)*	*Ratio (α/β)*
1	**20**	**10a**	**31**	0.2 eq. TMSOTf	DCM	-20	3	50	60/40
2	**20**	**A5**	-	0.1 eq. TMSOTf	DCM	-50	4	-	-
3	**21**	**10a**	**31**	0.3 eq. TMSOTf	DCM	-50	1.5	27	64/36
4	**21**	**30**	**35**	0.2 eq. TMSOTf	DCM	-50	2.5	24	68/32
5	**24**	**10a**	-	0.2 eq. TMSOTf	DCM	-50	2	-	-
6	**25**	**10a**	-	0.1 eq. TBDMSOTf	DCM	-40	4	-	-
7	**26**	**10a**	-	0.1 eq. TMSOTf	DCM	-30	2	-	-
8	**26**	**10a**	-	0.1 eq. TBDMSOTf	DCM	-30	4	-	-
9	**26**	**10a**	-	0.1 eq. TMSOTf	DCM	-50	1	-	-
10	**27**	**10a**	-	0.1 eq. $HClO_4$-SiO_2	DCM	-50	1	-	-
11	**27**	**30**	-	0.1 eq. TMSOTf	DCM	-80	3	-	-
12	**27**	**30**	-	0.3 eq. $HClO_4$-SiO_2	DCM	-80	2	-	-
13	**9**	**10a**	**32**	0.3 eq. TMSOTf	DCM	-50	2.5	30	65/35
14	**23**	**10a**	**32**	0.1 eq. TfOH	DCM	-50	1.5	64	63/73
15	**23**	**10a**	**32**	0.3 eq. TMSOTf	DCM	-50	2	49	79/21
16	**23**	**28**	**33**	0.2 eq. TMSOTf	DCM	-50	2	63	37/64
17	**23**	**29**	**34**	0.2 eq. TMSOTf	Diox. / DCM	-15	1.5	81	86/14
18	**23**	**30**	**36**	0.2 eq. TMSOTf	DCM	-80	1.5	66	91/9
19	**11**	**10b**	**13**	0.1 eq. PPh_3AuNTf_2	DCM	-30	O.N.	73	91/9

Experimental

General procedures

All chemicals were of commercial grade and were used as received unless stated otherwise. Solvents used in synthesis were dried and stored over 4Å molecular sieves, except for methanol, which was stored over 3 Å molecular sieves. All moisture/oxygen-sensitive reactions were performed under an argon or nitrogen atmosphere. Deuterated chloroform was stored over activated 3 Å molecular rods (rods, size 1/16 in., Sigma Aldrich) and potassium carbonate. Flash column chromatography was performed on silica gel 60 Å (0.04 – 0.063 mm, Screening Devices B.V.). TLC analysis was performed on TLC Silica gel 60 (Kieselgel 60 F254, Merck) with UV detection (254 nm) and by spraying with a solution of $(NH_4)_6Mo_7O_{24}\cdot H_2O$ (25 g/L), a solution of $(NH_4)_4Ce(SO_4)_4\cdot 2H_2O$ (10 g/L) in 10% sulfuric acid in water or with a solution of ninhydrin (3 g/L) in EtOH/AcOH (20:1 v/v) followed by charring at ± 150 °C. TLC-MS analysis was performed on a Camag TLC-MS Interface coupled with an API165 (SCIEX) mass spectrometer (eluted with *tert*-butylmethylether/EtOAc/MeOH, 5/4/1, v/v/v +0.1% formic acid, flow rate 0.12 mL/min). For LC−MS analysis, a JASCO HPLC system (detection simultaneously at 214 and 254 nm) equipped with an analytical C18 column (4.6 mm D × 50 mm L, 3 μ particle size) in combination with buffers A (H2O), B (MeCN), and C (0.5% aq TFA) and coupled to a PE/SCIEX API 165 single quadruple mass spectrometer (PerkinElmer) was used, unless stated otherwise. Alternatively, a Thermo Finnigan LCQ Advantage MAX ion-trap mass spectrometer with an electrospray ion source coupled to a Surveyor HPLC system (Thermo Finnegan) was used with the same analytical column. High-resolution mass spectra (HRMS) were recorded on a Waters Synapt G2-Si (TOF) equipped with an electrospray ion source in positive mode (source voltage 3.5 kV) and an internal lock mass LeuEnk ($M+H^+$ = 556.2771). ^1H and ^{13}C NMR spectra were recorded on a Bruker AV-400 NMR, a Bruker AV-500 NMR or a Bruker AV-850 NMR instrument. All samples were measured in $CDCl_3$, unless stated otherwise. Chemical shifts (δ) are given in ppm relative to tetramethyl silane as internal standard or the residual signal of the deuterated solvent. Coupling constants (*J*) are given in Hz. All given ^{13}C APT spectra are proton decoupled. NMR peak assignment was accomplished using COSY, HSQC. If necessary, additional NOESY, HMBC, HMBC-gated and HECADE experiments were used to further elucidate structures.

Synthetic procedures

Allyl-5-O-tertbutyldiphenylsilyl-2,3-bis-O-paramethoxybenzyl-α,β-D-ribofuranoside (2)

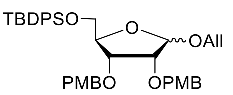

D-ribose (31.48 g, 210 mmol) was suspended in allyl alcohol (500 ml, 0.4 M). The suspension was cooled to 0 °C and acetyl chloride (3.0 ml, 42 mmol) was slowly added. The reaction mixture stirred over night at 4 °C. The reaction was quenched by adding Na_2CO_3. The reaction mixture filtrated, concentrated *in vacuo* and after coevaporation with toluene was dissolved in DCM (550 ml). Imidazole (21.10 g, 310 mmol), dried after coevaporation with toluene, was added and TBDPSCl (58.5 ml, 225 mmol) was slowly added. The reaction was then stirred for 1 hour and quenched by adding water. The reaction mixture was extracted with DCM and water, dried ($MgSO_4$), concentrated *in vacuo* and was dissolved in dry DMF (700 ml). The reaction was cooled to 0 °C and sodium hydride (472 mmol, 18.9 g, 60% dispersion in mineral oil) was added in small portions. After all sodium hydride has reacted, PMBCl (62.8 ml, 461 mmol) was slowly added and the reaction was allowed to warm up to room temperature. The reaction was stirred for 3 hours and then quenched by addition ice. The reaction mixture was diluted with diethylether and then partitioned between diethylether and water. The organic layer was washed with water, brine, dried ($MgSO_4$) and concentrated *in vacuo*. Column chromatography (pentane: EtOAc, 95/5 – 80/20) yielded the title compound (124.67 g, 186.55 mmol, 89% over three steps) as a clear oil. The analytical and physical data of the product were in agreement with literature[1].

(2S,3R,4R)-5-O-tert-butyldiphenylsilyl-2,3-bis-paramethoyxybenzyl-pentane-1,4-dimesylate (4)[2]

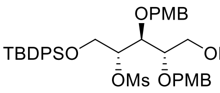

$[Ir(COD)(PPh_2Me)_2]PF_6$ (0.44 g, 0.52 mmol) was dissolved in freshly distilled THF (500 ml) and stirred under argon atmosphere. Hydrogen gas was bubbled through the solution until solution turns from red to yellow. The atmosphere was then replaced with argon

while making sure the solutions colour does not change back to red. A dropping funnel was charged with compound **2** (114.94 g, 172.0 mmol) dissolved in freshly distilled THF (900 ml), and slowly added to the catalyst in solution. The reaction was stirred over night on room temperature. Aqueous $NaHCO_3$ (sat.) solution (1 liter) and iodine (52.4 g, 206.4 mmol) was added and the reaction was stirred for 1.5 hrs. The reaction mixture was quenched by the addition of aq. $Na_2S_2O_3$ (sat.) and extracted with chloroform. The organic layer was dried ($MgSO_4$), concentrated *in vacuo*. Column chromatography (pentane: EtOAc, 90/10 – 70/30) yielded the title compound (72.96 g, 116.1 mmol, 68% (quantitative based on recovered starting material)).
The deallylated ribose (73 g, 116 mmol) was dissolved in methanol (460 ml) and sodium borohydride (8.8 g, 232 mmol) was added in small portions over a period of 30 minutes at 0 °C. The reaction was then stirred for 1,5 hour at room temperature. After these 1.5 hours the solvent was removed *in vacuo* and the residue was dissolved in EtOAc and washed with water and brine. The organic layer was dried ($MgSO_4$), concentrated *in vacuo* and dissolved in DCM (580 ml) and triethylamine (65 ml, 464 mmol). Mesyl chloride (27.2 ml, 348 mmol) was slowly added at -15 °C and the reaction was stirred for 45 minutes after which it was quenched upon addition of ice. The organic layer was washed with water, aq. $NaHCO_3$ (sat.), brine, dried ($MgSO_4$) and concentrated *in vacuo*. Column chromatography (pentane: EtOAc, 90/10 – 70/30) yielded the title compound (69.23 g, 88.0 mmol, 76% over two steps) as a clear oil. The product was not fully characterised but used directly in the next step.

^{1}H NMR (300 MHz, DMSO) δ 7.70 – 7.58 (m, 4H), 7.51 – 7.29 (m, 6H), 7.23 – 7.09 (m, 4H), 6.89 – 6.74 (m, 4H), 5.06 (dd, *J* = 6.7, 3.3 Hz, 1H), 4.66 – 4.38 (m, 4H), 4.42 – 4.22 (m, 2H), 4.04 – 3.91 (m, 1H), 3.87 – 3.66 (m, 9H), 2.98 (s, 3H), 2.91 (s, 3H), 1.06 (s, 9H).
^{13}C NMR (75 MHz, $CDCl_3$) δ 135.8, 135.7, 134.0, 130.3, 130.1, 130.1, 130.0, 129.1, 128.0, 114.0, 83.4, 76.4, 75.9, 73.3, 72.2, 67.9, 63.1, 55.4, 38.8, 37.7, 27.0, 19.3.

(2S,3R,4S)-5-O-tert-butyldiphenylsilyl-2,3-bis-paramethoyxybenzyl-1,4-di-bromopentane (5)[2]

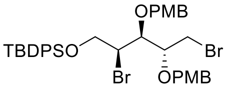

Compound **4** (66.58 g, 88.0 mmol), co-evaporated with toluene to remove traces of water, was dissolved in dry 2-butanone (440 ml). Lithium bromide (53 g, 616 mmol) was added and the reaction was refluxed for 3 nights. The reaction mixture was diluted with EtOAc and washed with water, aq. $NaHCO_3$ (sat.), brine, dried ($MgSO_4$) and concentrated *in vacuo*. The crude compound **5** was directly used in the synthesis of compound **6**.

5-O-tert-butyldiphenylsilyl-1,4-anhydro-2,3-bis-paramethoxybenzyl-4-thio-D-ribitol (6)[2]

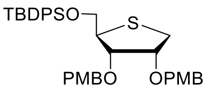

Compound **5** (88.0 mmol) and sodium sulfide nonahydrate (25.36 g, 105 mmol) were dissolved in DMF (300 ml). The reaction mixture was stirred at 100°C for 1 hour. Then the reaction mixture was diluted with EtOAc and washed with water, brine, dried ($MgSO_4$) and concentrated *in vacuo*. Column chromatography (pentane: EtOAc, 95/5 – 80/20) yielded the title compound (40.37 g, 64.2 mmol, 73% over 2 steps) as a clear oil.

^{1}H NMR (600 MHz, $CDCl_3$) δ: 7.63 (ddd, *J* = 8.1, 2.7, 1.4 Hz, 4H), 7.42 (dd, *J* = 7.5, 2.0 Hz, 2H), 7.37 (tt, *J* = 8.0, 1.0 Hz, 4H), 7.27 – 7.20 (m, 4H), 6.90 – 6.84 (m, 2H), 6.84 – 6.76 (m, 2H), 4.55 (d, *J* = 1.8 Hz, 2H), 4.44 (q, *J* = 9.1 Hz, 2H), 4.02 (t, *J* = 3.3 Hz, 1H), 3.93 (ddd, *J* = 7.6, 5.6, 3.4 Hz, 1H), 3.80 (s, 3H), 3.78 (s, 3H), 3.67 – 3.53 (m, 3H), 2.99 (AB, *J* = 7.7 Hz, 1H), 2.83 (AB, *J* = 5.6 Hz, 1H), 1.04 (s, 9H).
^{13}C NMR (151 MHz, $CDCl_3$) δ 159.3, 159.2, 135.8, 135.7, 133.4, 133.3, 130.4, 130.2, 129.4, 129.4, 127.9, 127.8, 113.9, 113.8, 80.0, 79.8, 71.7, 71.4, 66.0, 55.39, 55.37, 49.8, 30.6, 27.0, 19.4.
HRMS (ESI) m/z: $[M + Na]^+$: Calcd for $C_{37}H_{44}O_5SSiNa$ 651.2571; Found 651.2565

1-O-Acetyl-5-O-tert-butyldiphenylsilyl-2,3-bis-paramethoxybenzyl-4-thio-α,β-D-ribofuranoside (7)

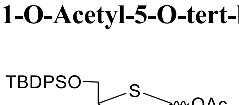

Compound **6** (7.68 mmol, 4.8 g) was dissolved in DCM (52 ml) and m-CPBA (8.06 mmol, 1.39 g) was added at -20 °C. The reaction mixture was stirred for 2 hours. The reaction mixture was quenched by the addition of aq. $Na_2S_2O_3$ (sat.) and the organic layer was washed with aq. $NaHCO_3$ (sat.). The organic layer was dried ($MgSO_4$), concentrated *in vacuo* and was dissolved in acetic anhydride (6 ml) and stirred at 100° for 3 hours. The reaction mixture was concentrated *in vacuo*. Column chromatography (pentane: EtOAc, 95/5 – 80/20) yielded the title compound (7.12 mmol, 4.89 g, 93% over two steps) as a clear oil.

^{1}H NMR (400 MHz, $CDCl_3$, α-anomer) δ: 7.59 (ddd, *J* = 7.9, 3.5, 1.5 Hz, 4H), 7.48 – 7.31 (m, 6H), 7.30 – 7.19 (m, 4H), 6.90 – 6.76 (m, 4H), 6.17 (d, *J* = 4.7 Hz, 1H), 4.70 – 4.41 (m, 4H), 4.14 (dd, *J* = 4.1, 2.0 Hz, 1H), 3.96 (t,

J = 4.4 Hz, 1H), 3.83 – 3.73 (m, 6H), 3.67 (ddd, J = 7.6, 5.5, 1.9 Hz, 1H), 3.55 (AB, J = 10.7, 5.5 Hz, 1H), 3.45 (AB, J = 10.7, 7.6 Hz, 1H), 2.13 (s, 3H), 1.02 (s, 9H).
^{13}C NMR (101 MHz, $CDCl_3$, α-anomer) δ: 171.0, 159.5, 159.2, 135.7, 135.6, 133.1, 133.1, 130.6, 123.0, 129.8, 129.4, 129.3, 127.9, 114.0, 113.8, 81.2, 80.1, 77.0, 72.6, 71.9, 65.6, 55.4, 50.9, 26.9, 21.6, 19.3.
^{1}H NMR (400 MHz, $CDCl_3$, β-anomer) δ: 7.66 (ddd, J = 5.4, 3.9, 1.8 Hz, 4H), 7.38 (dddd, J = 16.2, 8.4, 6.1, 1.6 Hz, 6H), 7.29 (d, J = 8.6 Hz, 2H), 7.14 (d, J = 8.6 Hz, 2H), 6.86 (d, J = 8.5 Hz, 2H), 6.80 (d, J = 8.6 Hz, 2H), 5.97 (d, J = 1.9 Hz, 1H), 4.67 (AB, J = 12.0 Hz, 1H), 4.54 (AB, J = 12.0 Hz, 1H), 4.42 – 4.33 (m, 2H), 4.12 (dd, J = 8.3, 3.5 Hz, 1H), 4.07 (dd, J = 3.5, 2.1 Hz, 1H), 3.84 (t, J = 4.6 Hz, 2H), 3.79 (s, 3H), 3.78 (s, 3H), 3.74 (dt, J = 8.7, 4.4 Hz, 1H), 2.02 (s, 3H), 1.05 (s, 9H).
^{13}C NMR (101 MHz, $CDCl_3$, β-anomer) δ: 170.0, 159.5, 159.4, 135.8, 135.7, 133.6, 133.2, 130.0, 129.8, 129.8, 129.7, 129.4, 127.8, 113.9, 113.9, 80.4, 80.1, 79.9, 72.5, 71.7, 63.5, 55.4, 50.4, 26.9, 21.3, 19.5.
HRMS (ESI) m/z: $[M + Na]^+$: Calcd for $C_{39}H_{46}O_7SSiNa$ 709.2626; Found 709.2626

5-O-tert-butyldiphenylsilyl -2,3-bis-paramethoxybenzyl-4-thio-α,β-D-ribofuranoside (8)

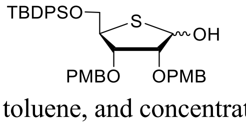

Compound **7** (7.12 mmol, 4.89 g) was dissolved in methanol (35 ml). The solution was cooled to 0 °C and sodium methoxide (3.56 mmol, 0.19 g) was added and the reaction was stirred for 2 hours. The reaction was quenched upon addition of acetic acid, diluted with toluene, and concentrated *in vacuo*. The residue was dissolved in DCM and washed with water. The organic layer was dried ($MgSO_4$), concentrated *in vacuo*. Column chromatography (pentane: EtOAc, 95/5 – 80/20) yielded the title compound (4.05 g, 6.30 mmol, 88%) as a clear oil.

^{1}H NMR (400 MHz, $CDCl_3$) δ 7.77 – 7.51 (m, 4H), 7.51 – 7.33 (m, 6H), 7.33 – 7.26 (m, 2H), 7.22 – 7.08 (m, 2H), 6.93 – 6.69 (m, 4H), 5.20 (dd, J = 5.8, 2.2 Hz, 1H), 4.60 (q, J = 12.0 Hz, 2H), 4.42 (d, J = 11.5 Hz, 1H), 4.33 (d, J = 11.5 Hz, 1H), 4.18 (dd, J = 7.6, 3.5 Hz, 1H), 4.08 – 3.97 (m, 1H), 3.90 – 3.64 (m, 9H), 2.16 (d, J = 5.8 Hz, 1H), 1.04 (s, 9H).
^{13}C NMR (101 MHz, $CDCl_3$) δ 159.5, 159.4, 135.8, 135.7, 135.7, 133.3, 133.2, 130.2, 130.0, 129.9, 129.9, 129.7, 129.6, 129.51, 129.46, 128.0, 127.93, 127.87, 114.0, 113.94, 113.90, 82.8, 80.3, 80.1, 72.5, 72.0, 64.3, 55.40, 55.38, 51.0, 27.0, 19.4
HRMS (ESI) m/z: $[M + Na]^+$: Calcd for $C_{37}H_{44}O_6SSiNa$ 667.2526; Found 667.2520

1-((N-Phenyl)-2,2,2-trifluoroacetimido)-5-O-tert-butyldiphenylsilyl-2,3-bis-paramethoxybenzyl-4-thio-β-D-ribofuranoside (9)

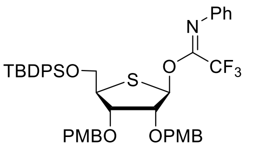

Compound **8** (0.32 mmol) and cesiumcarbonate (0.48 mmol, 0.010 g) were dissolved in acetone (3.5 ml) and water (0.1 ml). 2,2,2-trifluoro-*N*-phenylacetimidoyl chloride (0.48 mmol, 0.067 ml) was added and the reaction mixture was stirred at room temperature for 16 hours. The reaction mixture was filtered over a path of celite and concentrated *in vacuo*. Column chromatography, after neutralizing the silica with a triethylamine solution (pentane:triethylamine, 90/10), (pentane: EtOAc, 99/1 – 90/10) yielded the title compound (0.13 mmol, 0.106 g, 41% over two steps) as a clear oil.

^{1}H NMR (500 MHz, $CDCl_3$) δ: 7.72 – 7.63 (m, 4H), 7.47 – 7.32 (m, 6H), 7.29 (td, J = 8.2, 3.4 Hz, 4H), 7.19 (dd, J = 8.0, 6.1 Hz, 2H), 7.14 – 7.06 (m, 1H), 6.91 – 6.76 (m, 6H), 5.98 (s, 1H), 4.67 – 4.55 (m, 2H), 4.52 – 4.40 (m, 2H), 4.25 – 4.08 (m, 2H), 3.91 – 3.81 (m, 2H), 3.81 – 3.72 (m, 7H), 1.06 (s, 9H).
^{13}C NMR (126 MHz, $CDCl_3$) δ: 159.5, 143.8, 135.9, 135.7, 133.6, 133.2, 129.8, 129.8, 129.7, 129.6, 128.8, 127.8, 124.3, 120.6, 119.6, 113.9, 84.1, 80.5, 79.9, 72.8, 72.1, 63.6, 55.3, 50.2, 26.8, 19.4.
HRMS (ESI) m/z: $[M + H]^+$: Calcd for $C_{45}H_{48}F_3NO_6SSiH$ 816.2996; Found 816.2999

(3R,4S,5R)-5-(((tert-butyldiphenylsilyl)oxy)methyl)-3,4-bis((4-methoxybenzyl)oxy)tetrahydrothiophen-2-yl 2-(hex-1-yn-1-yl)benzoate (11)

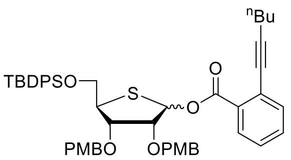

Compound **8** (1.44 mmol, 0.93 g) was dissolved in DCM (8 ml) and DiPEA (12.96 mmol, 1.68 g, 2.26 ml), DMAP (1.44 mmol, 0.18 g) and EDCI·HCl (4.32 mmol, 0.83 g). Next, a solution of ortho-hexynylbenzoic acid (4.32 mmol, 0.87 g) in DCM (7 ml) was added to the reaction mixture and it was stirred overnight on room temperature. The reaction mixture was washed with with aq. $NaHCO_3$ (sat.), dried

(Na_2SO_4) and concentrated *in vacuo*. Column chromatography (pentane: EtOAc, 95/5 – 85/15) yielded the title compound (1.44 mmol, 1.19 g, quant.) as a clear oil.

^{1}H NMR (300 MHz, $CDCl_3$) δ 7.78 – 7.63 (m, 1H), 7.69 – 7.58 (m, 4H), 7.56 – 7.07 (m, 13H), 6.94 – 6.73 (m, 4H), 6.25 (d, *J* = 1.7 Hz, 1H), 4.76 (d, *J* = 12.1 Hz, 1H), 4.69 – 4.53 (m, 1H), 4.51 – 4.35 (m, 1H), 4.41 – 4.29 (m, 1H), 4.27 – 4.15 (m, 1H), 4.20 – 4.02 (m, 1H), 3.95 – 3.87 (m, 3H), 3.79 (s, 3H), 3.77 (s, 3H), 2.41 (t, *J* = 7.0 Hz, 2H), 1.58 (s, 2H), 1.68 – 1.39 (m, 2H), 0.99 – 0.88 (m, 12H).
^{13}C NMR (75 MHz, $CDCl_3$) δ 135.9, 135.8, 134.6, 132.0, 130.6, 129.9, 129.8, 129.5, 127.81, 127.79, 127.1, 114.0, 113.9, 80.5, 80.4, 80.0, 72.5, 71.7, 63.8, 55.4, 50.4, 30.9, 26.8, 22.2, 19.7, 19.4.
HRMS (ESI) m/z: $[M + Na]^+$: Calcd for $C_{50}H_{56}O_7SSiNa$ 851.3408; Found 851.3400

(E)-prop-1-en-1-yl N-(((9H-fluoren-9-yl)methoxy)carbonyl)-O-((2S,3R,4S,5R)-5-(((tert-butyldiphenylsilyl)oxy)methyl)-3,4-bis((4-methoxybenzyl)oxy)tetrahydrothiophen-2-yl)-L-serinate (13)

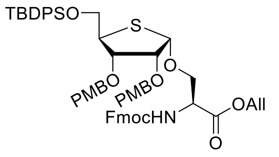

Donor **11** (0.09 mmol, 0.083 g) and acceptor **A2** (0.135 mmol, 0.050 g), dried by co-evaporating with dry toluene thrice, were dissolved in DCM (1.8 ml). Freshly dried molecular sieves (rods) were added. After stirring for 30 mins the reaction was cooled to -30 °C, next freshly prepared PPh_3AuNTf_2 (0.1M in DCM) solution was added (0.1 ml). The reaction mixture was stirred overnight at -30 °C. The reaction mixture was filtered over a pad of celite and concentrated *in vacuo*. Column chromatography (pentane: EtOAc, 95/5 – 75/25) yielded the title compound (0.066 mmol, 0.065 g, 73%) as a clear oil.

^{1}H NMR (400 MHz, $CDCl_3$) δ 7.74 (ddd, *J* = 15.7, 7.5, 3.1 Hz, 3H), 7.72 – 7.46 (m, 6H), 7.46 – 7.29 (m, 9H), 7.31 – 7.20 (m, 4H), 6.89 – 6.83 (m, 2H), 6.75 (dd, *J* = 15.7, 8.5 Hz, 2H), 6.39 (d, *J* = 8.8 Hz, 1H), 5.87 (ddt, *J* = 17.3, 10.7, 5.5 Hz, 1H), 5.34 – 5.18 (m, 1H), 5.17 – 5.08 (m, 2H), 4.71 – 4.50 (m, 4H), 4.50 – 4.36 (m, 3H), 4.29 – 4.12 (m, 3H), 4.04 (dd, *J* = 9.1, 3.5 Hz, 1H), 4.01 – 3.90 (m, 1H), 3.86 (t, *J* = 4.2 Hz, 1H), 3.78 (s, 3H), 3.67 (s, 3H), 3.68 – 3.51 (m, 2H), 3.46 (dd, *J* = 10.4, 7.6 Hz, 1H), 1.03 (s, 9H).
^{13}C NMR (101 MHz, $CDCl_3$) δ 170.4, 159.4, 159.1, 156.5, 144.3, 143.9, 141.4, 141.3, 135.8, 135.7, 135.7, 133.1, 133.1, 131.9, 130.5, 130.1, 130.0, 129.8, 129.6, 129.5, 129.3, 127.90, 127.88, 127.8, 127.70, 127.66, 127.2, 127.1, 125.6, 125.3, 120.1, 120.0, 119.9, 118.4, 113.9, 113.8, 113.7, 85.0, 82.6, 80.1, 72.2, 72.0, 67.9, 67.3, 67.1, 66.1, 65.8, 55.4, 55.3, 55.2, 54.3, 50.9, 47.2, 26.93, 26.87, 19.4.
HRMS (ESI) m/z: $[M + Na]^+$: Calcd for $C_{58}H_{63}NO_{10}SSiNa$ 1016.3834; Found 1016.3837

N-(((9H-fluoren-9-yl)methoxy)carbonyl)-O-((2S,3R,4S,5R)-5-(((tert-butyldiphenylsilyl)oxy)methyl)-3,4-bis((4-methoxybenzyl)oxy)tetrahydrothiophen-2-yl)-L-serine (1)

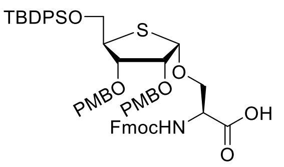

Compound **13** (0.24 mmol, 0.24 g) and DMBA (0.36 mmol, 0.056 g) were dissolved in DCM (2.4 ml). $Pd(PPh_3)_4$ (0.024 mmol, 0.028 g) was added and the reaction mixture was stirred on room temperature for 1 hour. After completion of the reaction THT (2.4 mmol, 0.21 g, 0.21 ml) was added, the reaction mixture was diluted with DCM and washed with a 1M HCl (aq.) solution. The reaction mixture was filtered over a pad of celite and concentrated *in vacuo*. Column chromatography (DCM: MeOH, 99/1 – 90/10) followed by size exclusion column chromatography yielded the title compound (0.16 mmol, 0.157 g, 68%) as a clear oil.

^{1}H NMR (400 MHz, $CDCl_3$) δ 7.68 (d, *J* = 7.5 Hz, 2H), 7.53 (d, *J* = 7.4 Hz, 6H), 7.41 (dd, *J* = 9.6, 5.6 Hz, 2H), 7.33 (t, *J* = 7.8 Hz, 6H), 7.26 – 7.10 (m, 7H), 6.79 – 6.68 (m, 4H), 6.24 (s, 1H), 4.90 (s, 1H), 4.61 – 4.56 (m, 1H), 4.49 (d, *J* = 11.9 Hz, 2H), 4.39 (s, 1H), 4.32 – 4.21 (m, 2H), 4.13 (t, *J* = 7.1 Hz, 1H), 4.04 (s, 1H), 3.93 (s, 1H), 3.82 – 3.73 (m, 2H), 3.73 – 3.60 (m, 6H), 3.53 (t, *J* = 6.1 Hz, 1H), 3.43 (s, 2H), 3.35 – 3.26 (m, 1H), 0.97 (s, 9H)
^{13}C NMR (126 MHz, $CDCl_3$) δ 141.4, 135.7, 135.7, 133.3, 130.2, 130.1, 129.9, 128.3, 128.0, 127.9, 127.7, 127.2, 125.4, 120.0, 114.1, 114.0, 87.0, 81.0, 79.0, 72.0, 71.5, 69.0, 67.0, 65.2, 55.28, 55.26, 54.0, 50.0, 47.3, 27.0, 19.4
HRMS (ESI) m/z: $[M + Na]^+$: Calcd for $C_{55}H_{59}NO_{10}SSiH$ 954.3702; Found 954.3702

Solid phase synthesis

Peptide synthesis

The intermediate peptides were synthesized using standard, Fmoc-based solid phase peptide synthesis utilizing (pre-loaded) Tentagel® S AC purchased from Rapp Polymer Gmbh. Coupling cycles were as followed: Fmoc deprotection: 2x2 min, 1x5 min treatment with 20% piperidine in DMF. Coupling: treatment of 6 eq. amino acid, 6 eq. HCTU (0.25M in DMF) and 12 eq. DIPEA (1M in DMF) for 30 minutes. Capping: 2x2 min treatment of the resin with a 10% Ac_2O solution in DMF and catalytic DIPEA. Washing between the steps was done with DMF. Ribosylated amino acids **28**, **29** and **30** were incorporated in the sequence by adding a solution of 2 eq. building block in a 0.25M HCTU solution (2 eq.) in DMF and a 1M DIPEA solution (4 eq.) in DMF to the resin in a fritted syringe. The resin was shaken overnight and thoroughly washed.

On-resin phosphorylation

The resin was treated with a sufficient amount of 1M TBAF in THF for 30 minutes. The resin was thoroughly washed with DCM and DMF before the treatment was repeated once, furnishing the desilylated intermediate. The resin was then extensively washed with dry MeCN and flushed with nitrogen to remove traces of water before the resin was subjected to a solution of 5 eq. of $(FmO)_2PN(iPr)_2$ (0.25M in MeCN) with 10 eq. ETT solution (0.25M in MeCN). The resin was shaken for 30 minutes after which the resin was washed with MeCN. The resin was then treated with a sufficient amount of CSO solution (0.5M in MeCN) for 30 minutes. The resin was then treated with a 10% DBU solution in DMF (2x 15 minutes) to furnish the crude, immobilized and deprotected phosphoribosylated peptide.

Construction of the pyrophosphates

The resin was extensively washed with dry MeCN and flushed with nitrogen to remove traces of water. The resin was then treated with a solution of 5'-*O*-(N^6-*tert*-butyloxycarbonyl-2',3'-di-*O*-*tert*-butyldimethylsilyladenosine)-2-cyanoethyl-*N*-*N*-diisopropylphosphoramidite (3 eq., 0.3M in MeCN) and ETT (6 eq., 0.25M in MeCN) for 30 minutes. The resin was thoroughly washed with MeCN before a sufficient amount of CSO (0.5M in MeCN) was added to the resin and shaken for 30 minutes.

Final deprotection and cleavage

The resin was treated with a 10% DBU solution in DMF (2x 10 minutes) to remove the cyano ethyl protecting group. The resin was then treated with a 1M TBAF solution in THF (2x 45 minutes) and washed with DMF followed by DCM. Final cleavage/deprotection occurred by treating the resin with a cleavage cocktail (2.5/10/87.5 TIS/TFA/DCM) for 4 hours. The crude products were collected by filtration and the resin was washed with a solution of 1/1/1 water/*t*BuOH/MeCN. The solvents were evaporated *in vacuo* and co-evaporated with a 1/1/1 water/*t*BuOH/MeCN solution.

Ac-Pro-Ala-Lys-Ser(5-O-adenosine diphosphate-α-D-4-thio-ribosyl)-Ala-Pro-Ala-Pro-Lys-Lys-Gly-OH (19)

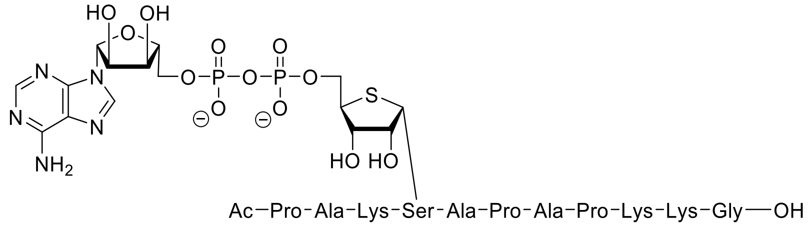

The general procedures described above were applied to 50 µmol Tentagel® S AC resin preloaded with glycine. The amino acids used were: Fmoc-Pro-OH, Fmoc-Ala-OH, Fmoc-Lys(Mtt)-OH and **2**. Oxidation steps were carried out with a 0.5M *t*BuOOH solution in MeCN. The crude peptide was purified by RP-HPLC in NH_4OAc buffer. The pure fractions were concentrated, co-evaporated extensively with a 1:1 mixture of MeCN:Milli-Q water, redissolved in Milli-Q water and lyophilized to obtain titled compound as a white solid (3.43 mg, 2.04 µmol, 4.1%).

^{1}H NMR (850 MHz, D_2O) δ 8.49 (s, 1H, H-2 adenine), 8.24 (s, 1H, H-8 adenine), 6.10 (d, J = 8.5 Hz, 1H, H-1' adenosine).

^{31}P NMR (202 MHz, D_2O) δ -10.72, -10.83, -11.04, -11.14.

LC-MS (0 -> 50% B in A): Rt = 4.42.

HRMS (ESI) m/z: $[M + 2H]^{2+}$: Calcd for $C_{64}H_{105}N_{19}O_{26}P_2S2H$ 825.8410; Found 825.8408

Expression plasmids and protein purification

The construction of the expression plasmids and the purification procedures were described earlier.[3,4,5] Briefly, expression plasmids were transferred into Rossetta (DE3) cells and grown at 37 °C to an OD_{600} of 0.6 in LB medium supplemented with 1% (w/v) D-glucose and appropriate antibiotics. For (ADP-ribosyl)hydrolases (ARH1, ARH2, and ARH3) the medium was further enriched by addition of 2 mM $MgSO_4$. Expression was induced with 0.4 mM isopropyl-β-D-1-thioglactopyranoside (IPTG) and cultured were allowed to grow overnight at 17 °C. Cultures were harvested by centrifugation, pellets resuspended in lysis buffer (50 mM TrisHCl [pH 8], 500 mM NaCl and 25 mM imidazole) and stored at -20 °C until use. Proteins were purified by Ni^{2+}-NTA chromatography (Jena Bioscience) according to the manufacturer's protocol using the following buffers: all buffers contained 50 mM TrisHCl (pH 8) and 500 mM NaCl; additionally, the lysis buffer contained 25 mM, the washing buffer 40 mM, and the elution buffer 500 mM imidazole. Proteins were dialyzed overnight against 50 mM TrisHCl (pH 8), 200 mM NaCl, 1 mM dithiothreitol and 5% (v/v) glycerol and stored at -80 °C. For the purification of ARH1, ARH2, and ARH3 all purification buffers were additionally supplemented with 10 mM $MgCl_2$.

(ADP-ribosyl)hydrolase activity assay

The peptide demodification assay was described earlier.[6,7] Briefly, peptide concentrations for the assay were estimated using absorbance at λ_{260nm} using the molar extinction coefficient of ADP-ribose (13,400 M^{-1} cm^{-1}). 10 μM indicated peptide were demodified by incubation with 0.5 μM hydrolase for 60 min at 30 °C in assay buffer (50 mM TrisHCl [pH 8], 200 mM NaCl, 10 mM $MgCl_2$, 1 mM dithiothreitol and 0.2 μM human NUDT5 [REF]).[6] Reactions were stopped and analysed by performing the AMP-Glo™ assay (Promega) according to the manufacturer's protocol. Luminescence was recorded on a SpectraMax M5 plate reader (Molecular Devices) and data analysed with GraphPad Prism 7. Control reactions were carried out in absence of peptide.

References

1. Kistemaker, H. A.; Nardozza, A. P.; Overkleeft, H. S.; van der Marel, G. A.; Ladurner, A. G.; Filippov, D. V. Synthesis and Macrodomain Binding of Mono-ADP-Ribosylated Peptides. *Angew. Chem. Int. Ed. Engl.* **2016,** *55* (36), 10634-10638.

2. Minakawa, N.; Kato, Y.; Uetake, K.; Kaga, D.; Matsuda, A. An improved large scale synthesis of 1,4-anhydro-4-thio-D-ribitol. *Tetrahedron* **2003**, *59*, 1699-1702.

3. Fontana, P.; Bonfiglio, J. J.; Palazzo, L.; Bartlett, E.; Matic, I.; Ahel, I. Serine ADP-ribosylation reversal by the hydrolase ARH3. *Elife* **2017**, *6*: e28533

4. Palazzo, L.; Thomas, B.; Jemth, A. S.; Colby, T.; Leidecker, O.; Feijs, K. L. H.; Zaja, R.; Loseva, O.; Puigvert, J. C.; Matic, I.; Helleday, T; Ahel, I. Processing of protein ADP-ribosylation by Nudix hydrolases. *Biochem. J.* **2015**, *468*, 293-301.

5. Slade, D.; Dunstan, M. S.; Barkauskaite, E.; Weston, R.; Lafite, P.; Dixon, N.; Ahel, M.; Leys, D.; Ahel, I. The structure and catalytic mechanism of a poly(ADP-ribose) glycohydrolase. *Nature* **2011**, *477*, 616-620.

6. Voorneveld, J.; Rack, J. G. M.; Ahel, I.; Overkleeft, H. S.; van der Marel, G. A.; Filippov, D. V. Synthetic alpha- and beta-Ser-ADP-ribosylated Peptides Reveal alpha-Ser-ADPr as the Native Epimer. *Org. Lett.* **2018**, *20*, 4140-4143.

7. Rack, J. G. M.; Ahel, I. A Simple Method to Study ADP-Ribosylation Reversal: From Function to Drug Discovery. *Methods Mol. Biol.* **2023**, *2609*, 111-132.

NMR Spectra

(2S,3R,4R)-5-O-tert-butyldiphenylsilyl-2,3-bis-paramethoyxybenzyl-pentane-1,4-dimesylate (4)

^{1}H-NMR, $CDCl_3$, 300 MHz

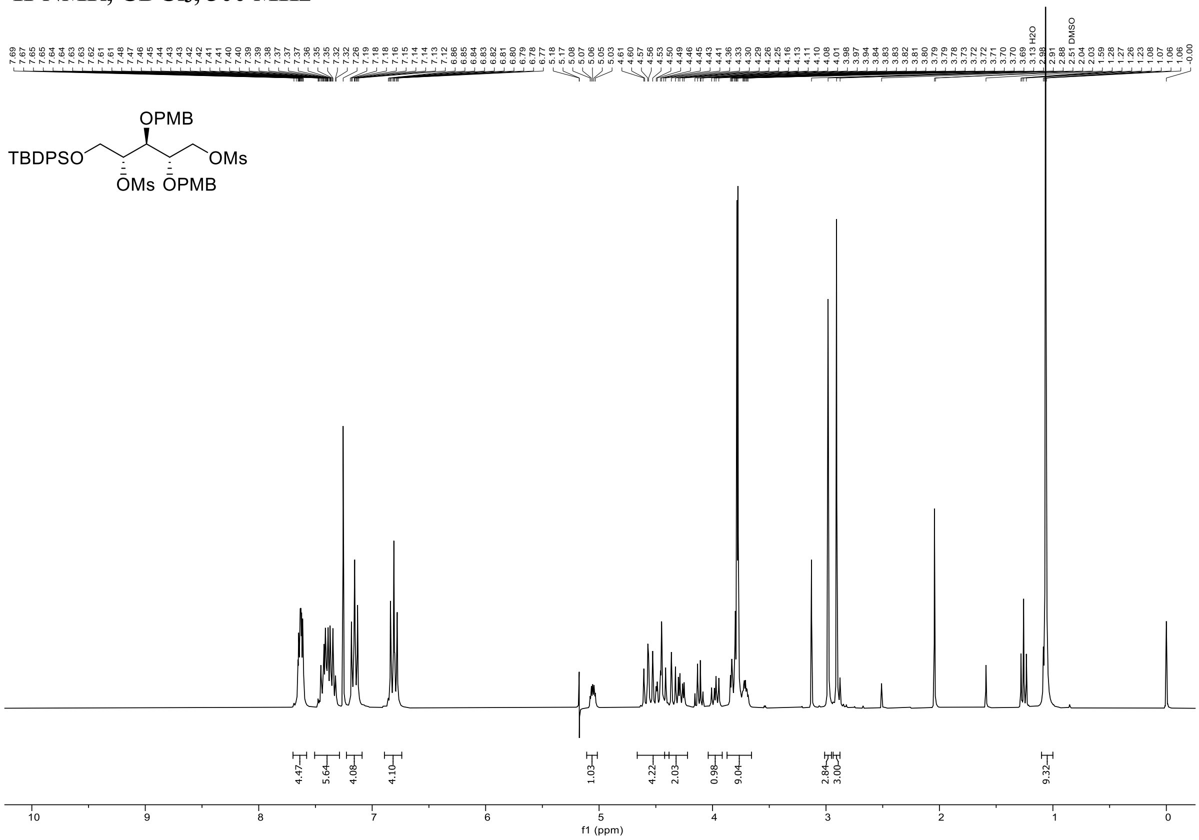

^{13}C-NMR, $CDCl_3$, 101 MHz

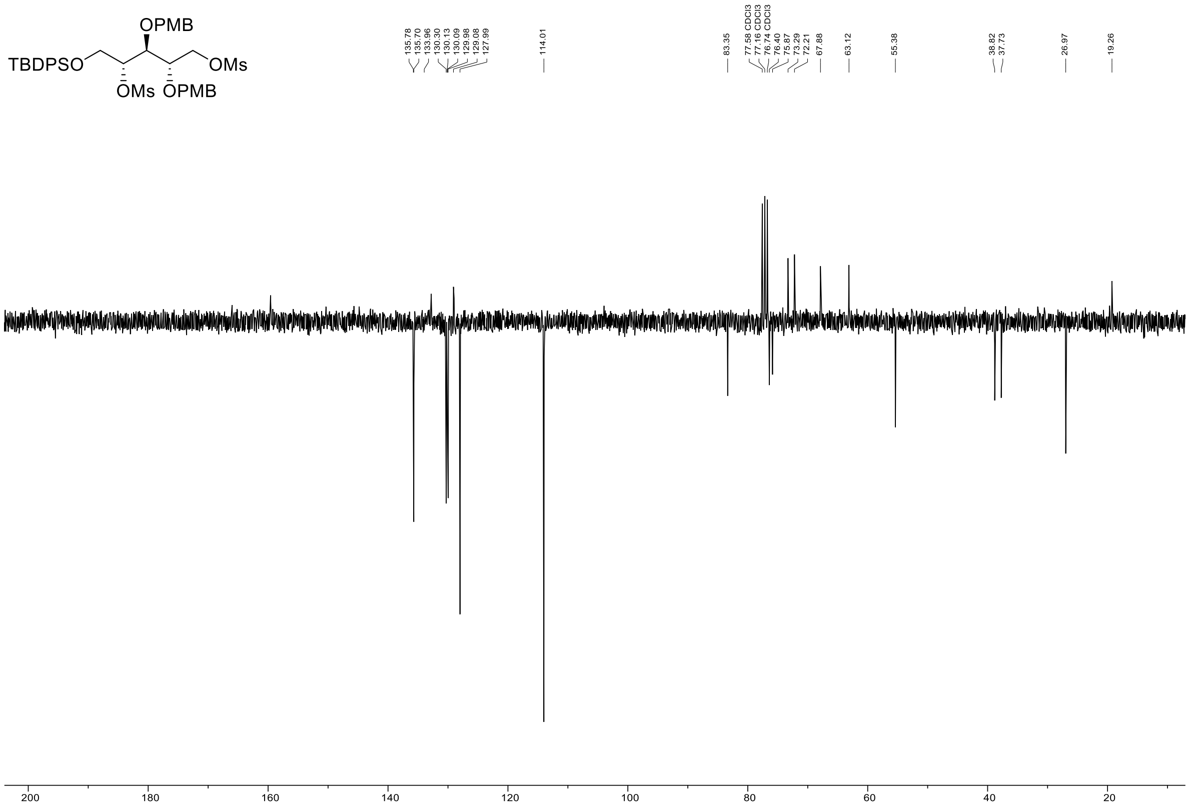

5-O-tert-butyldiphenylsilyl-1,4-anhydro-2,3-bis-paramethoxybenzyl-4-thio-D-ribitol (6)

^{1}H-NMR, $CDCl_3$, 600 MHz

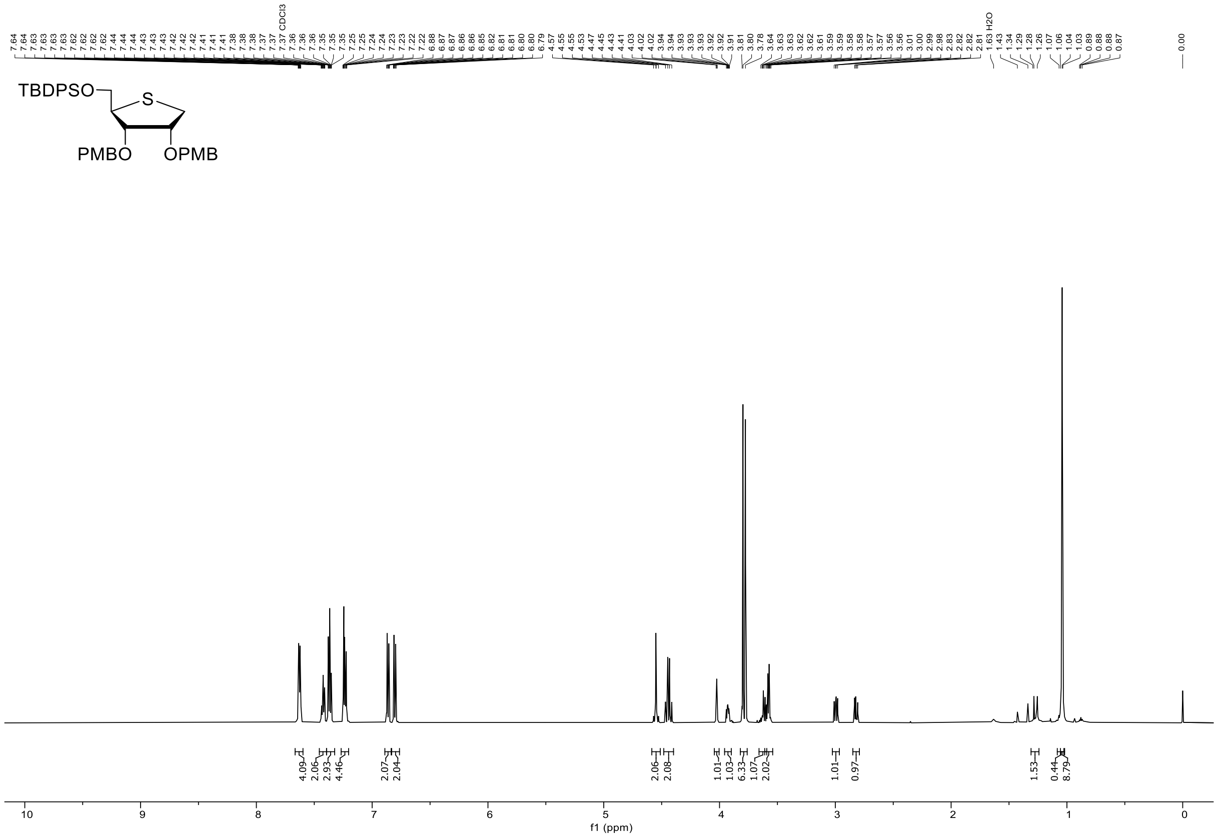

^{13}C-NMR, $CDCl_3$, 151 MHz

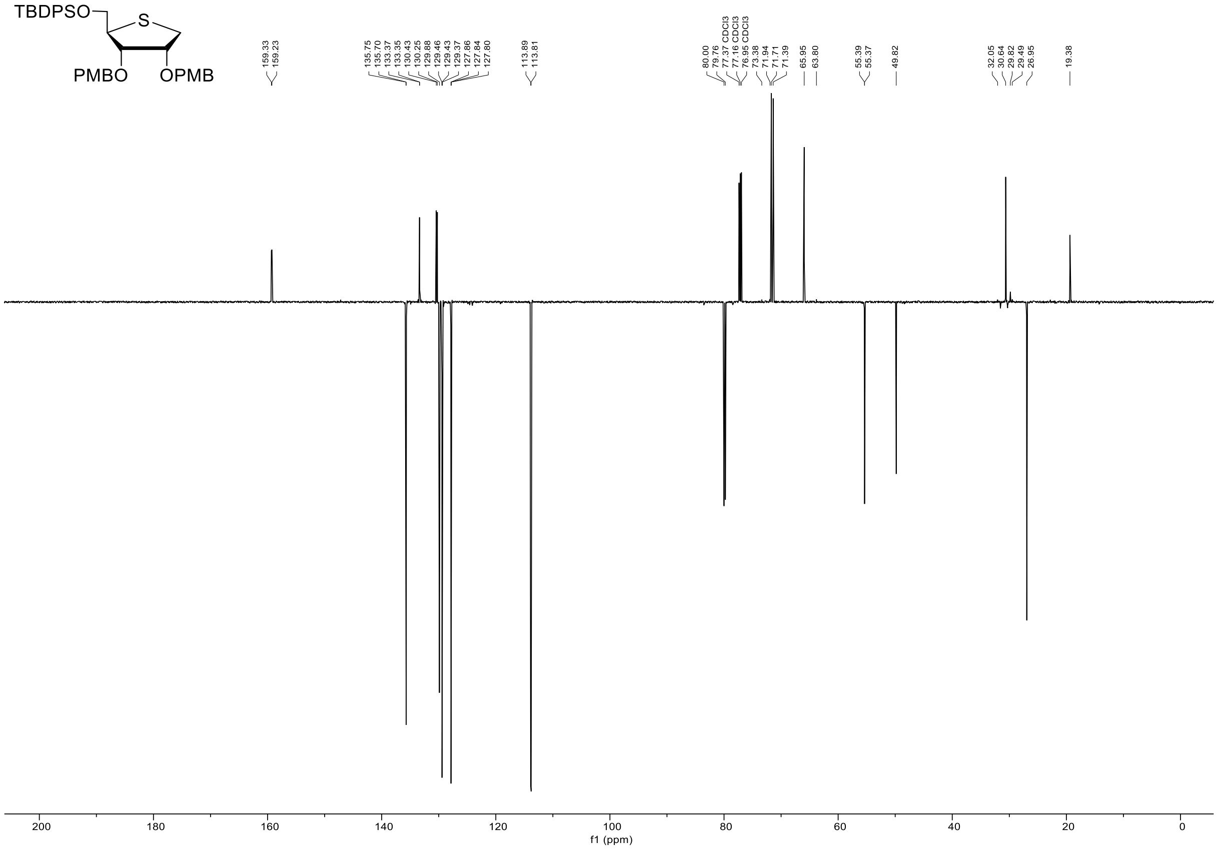

1-O-Acetyl-5-O-tert-butyldiphenylsilyl-2,3-bis-paramethoxybenzyl-4-thio-α,β-D-ribofuranoside (7)

^{1}H-NMR, $CDCl_3$, 400 MHz

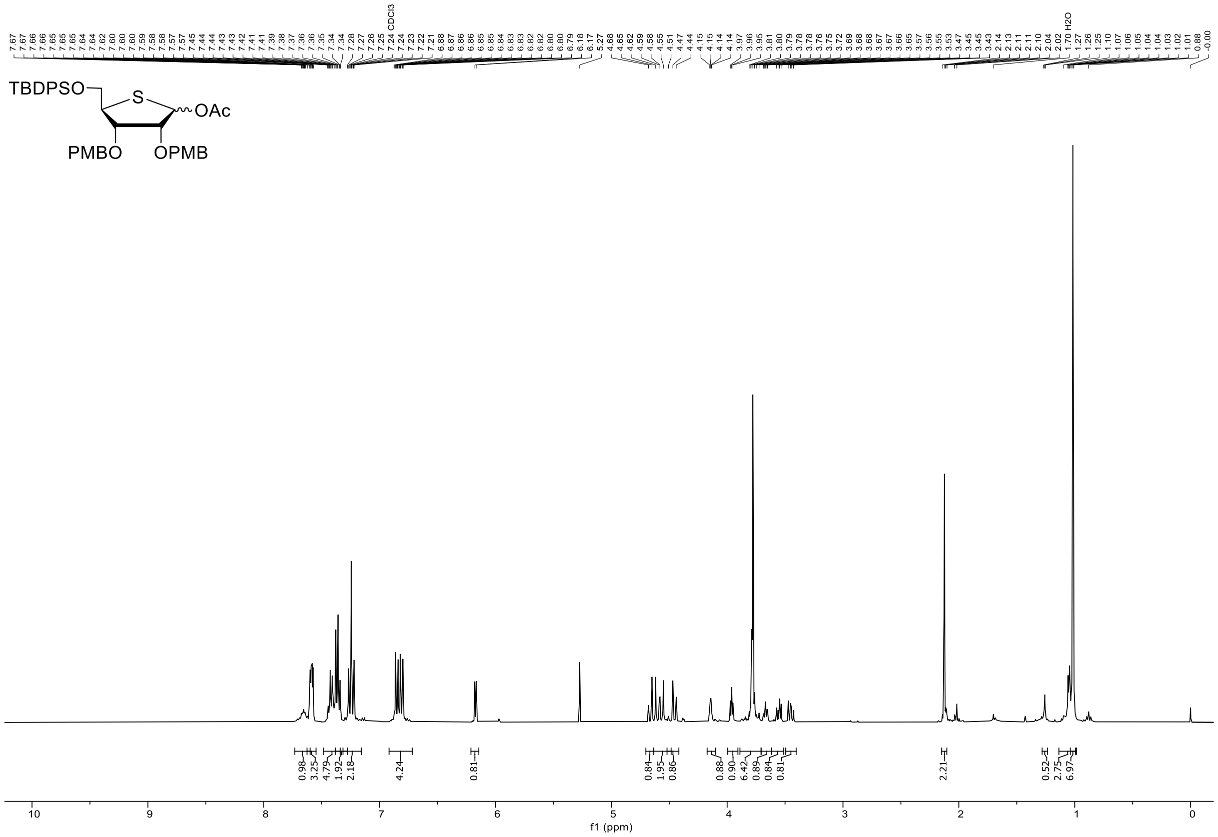

^{13}C-NMR, $CDCl_3$, 101 MHz

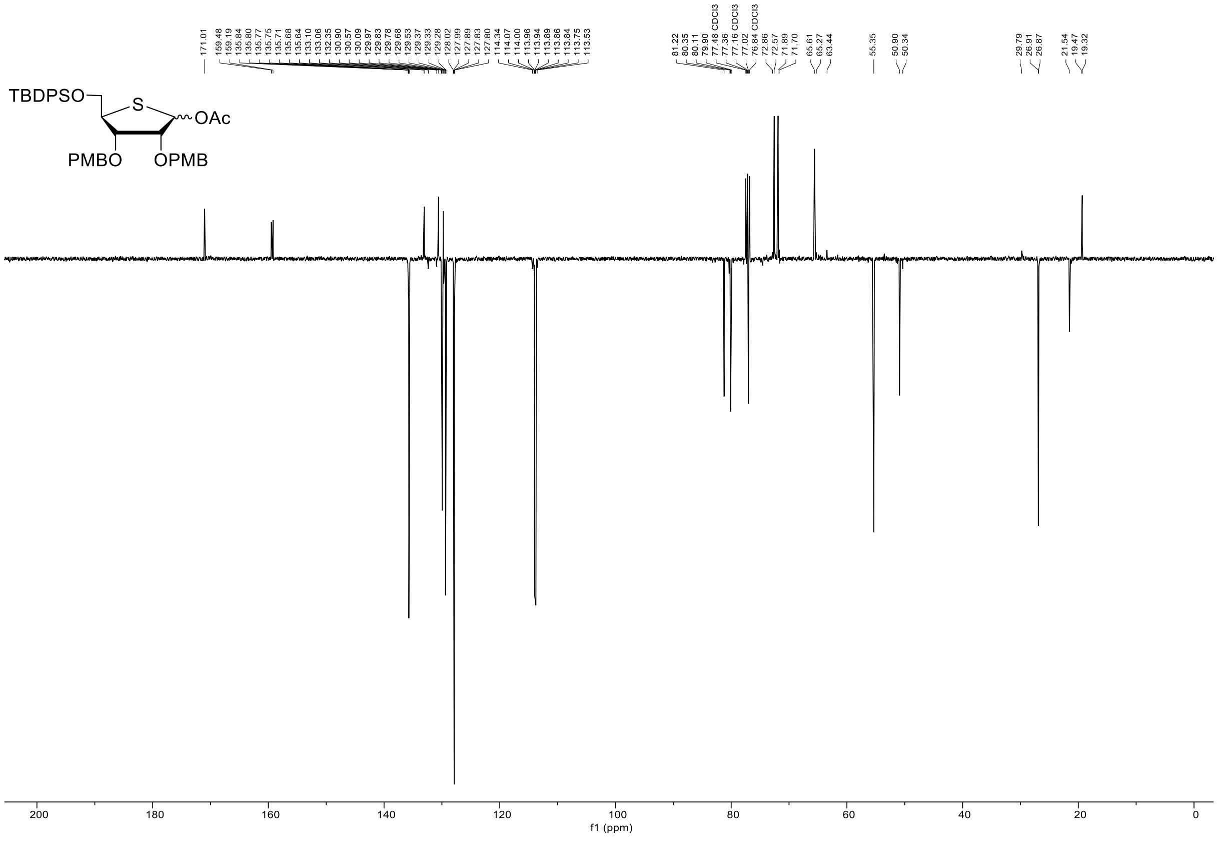

5-O-tert-butyldiphenylsilyl -2,3-bis-paramethoxybenzyl-4-thio-α,β-D-ribofuranoside (8)
^{1}H-NMR, CDCl₃, 400 MHz

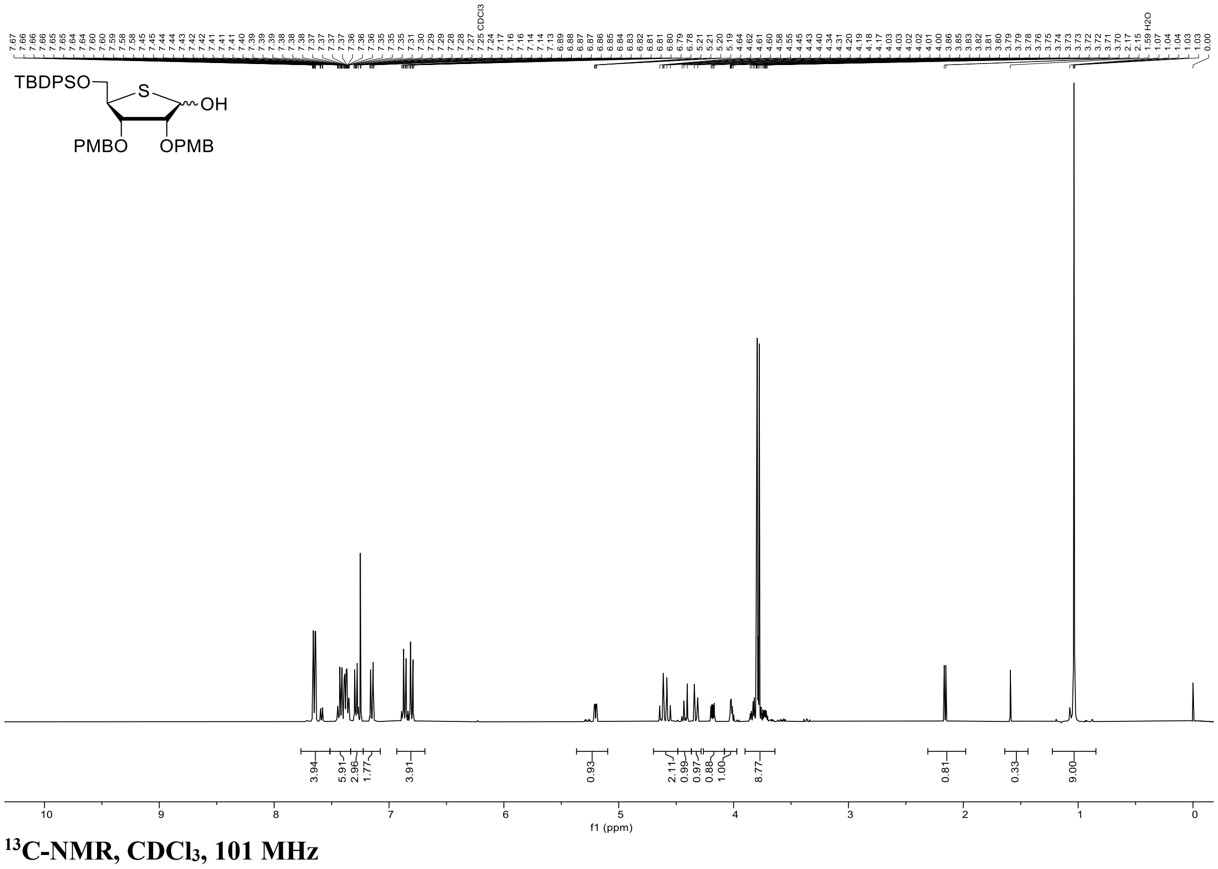

^{13}C-NMR, CDCl₃, 101 MHz

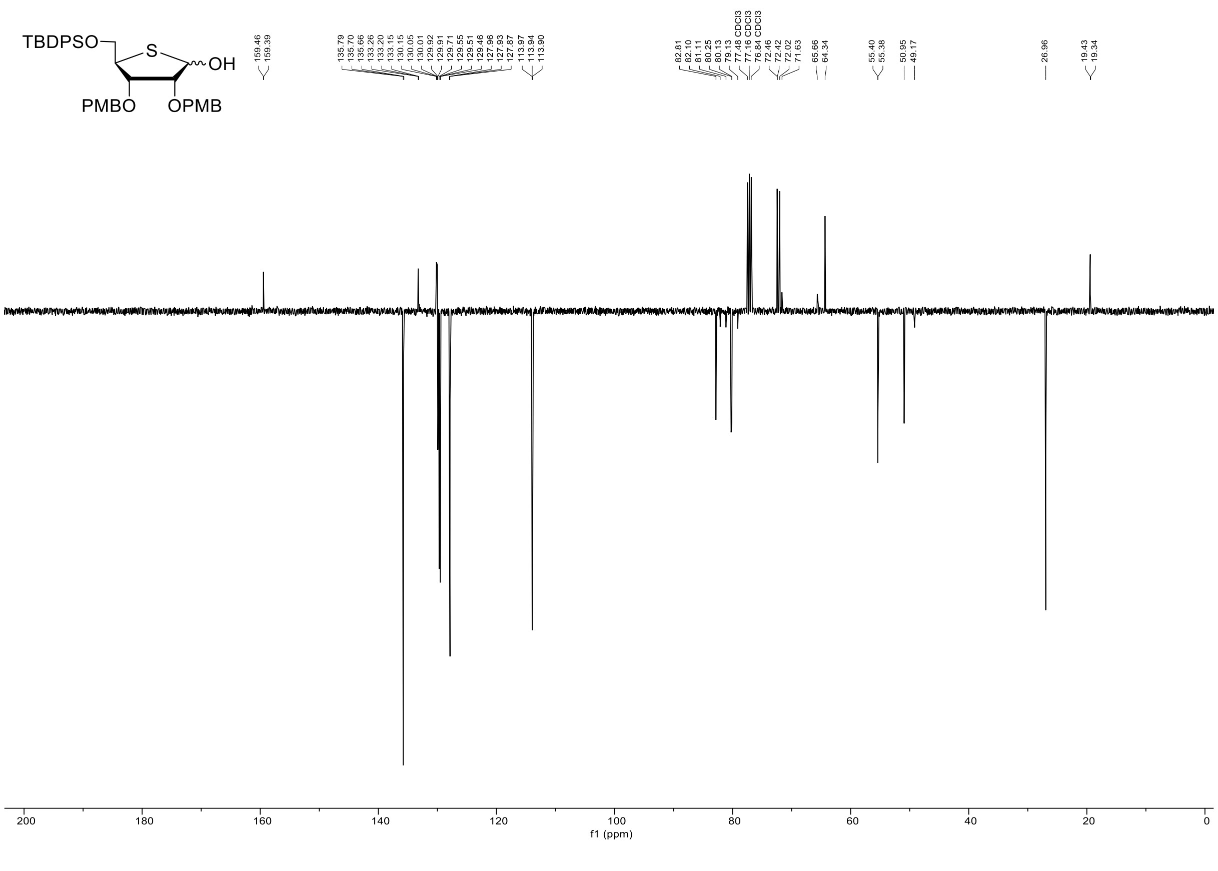

1-((N-Phenyl)-2,2,2-trifluoroacetimido)-5-O-tert-butyldiphenylsilyl-2,3-bis-paramethoxybenzyl-4-thio-β-D-ribofuranoside (9)

^{1}H-NMR, $CDCl_3$, 500 MHz

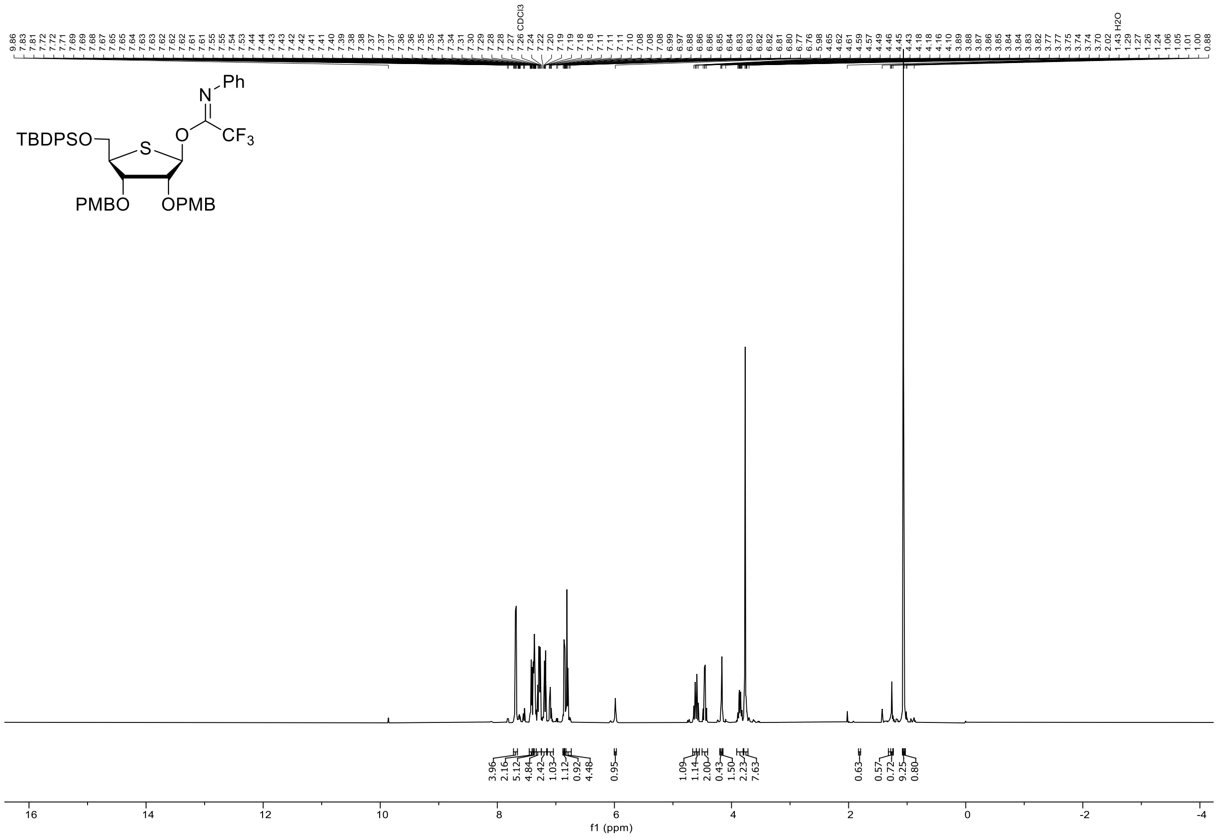

^{13}C-NMR, $CDCl_3$, 126 MHz

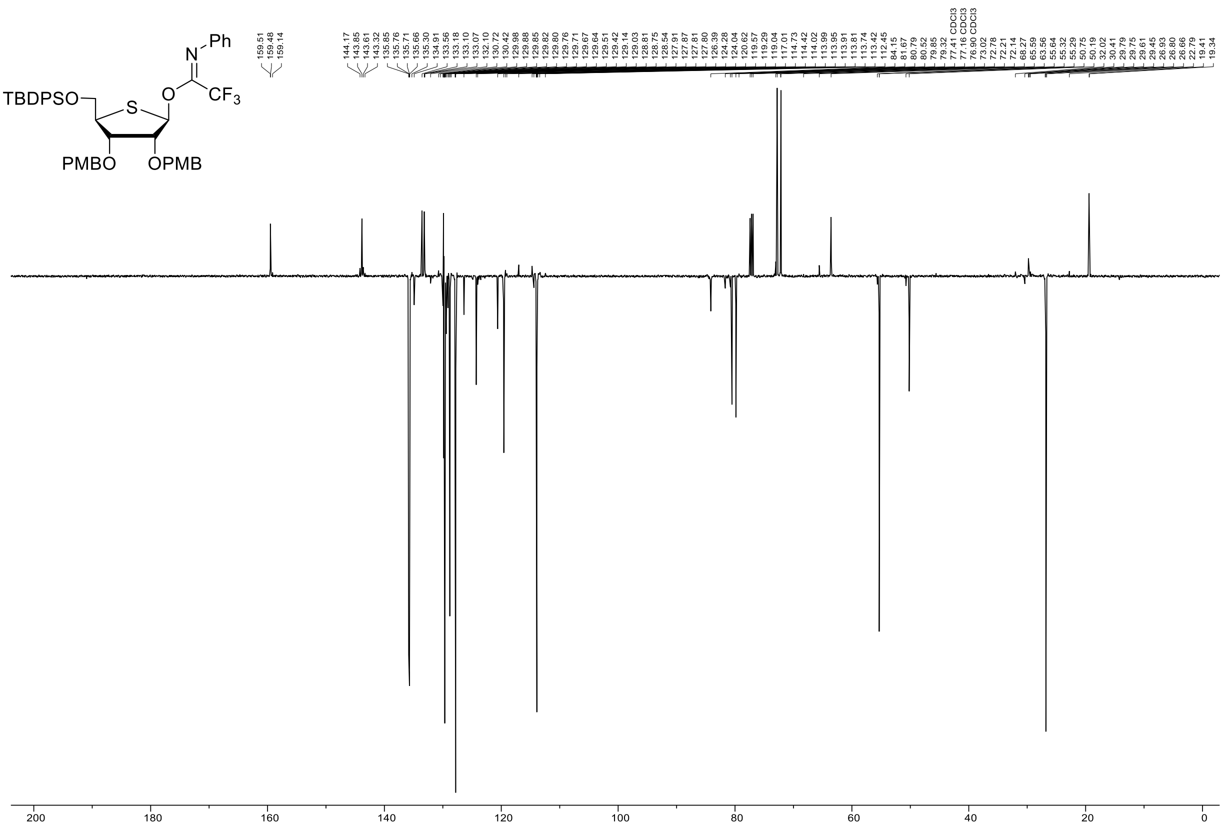

(3R,4S,5R)-5-(((tert-butyldiphenylsilyl)oxy)methyl)-3,4-bis((4-methoxybenzyl)oxy)tetrahydrothiophen-2-yl 2-(hex-1-yn-1-yl)benzoate (11)

^{1}H-NMR, CDCl$_3$, 300 MHz

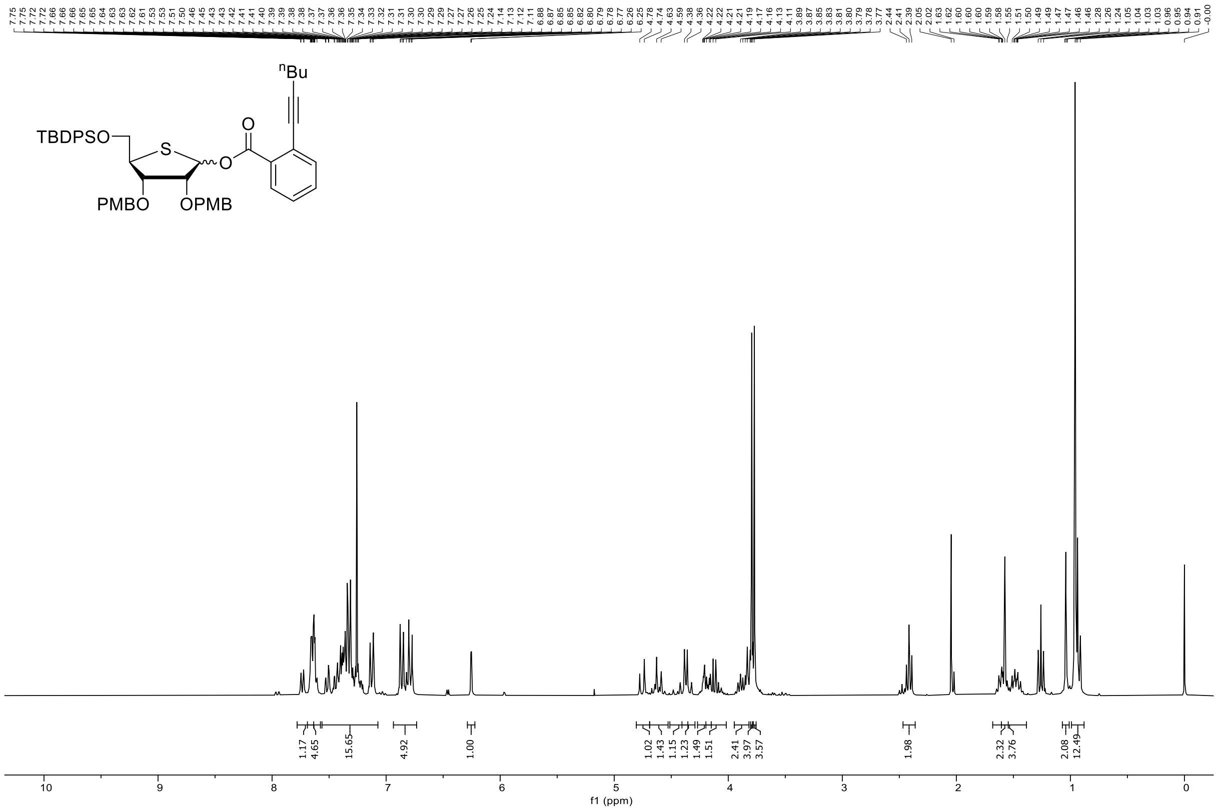

^{13}C-NMR, CDCl$_3$, 101 MHz

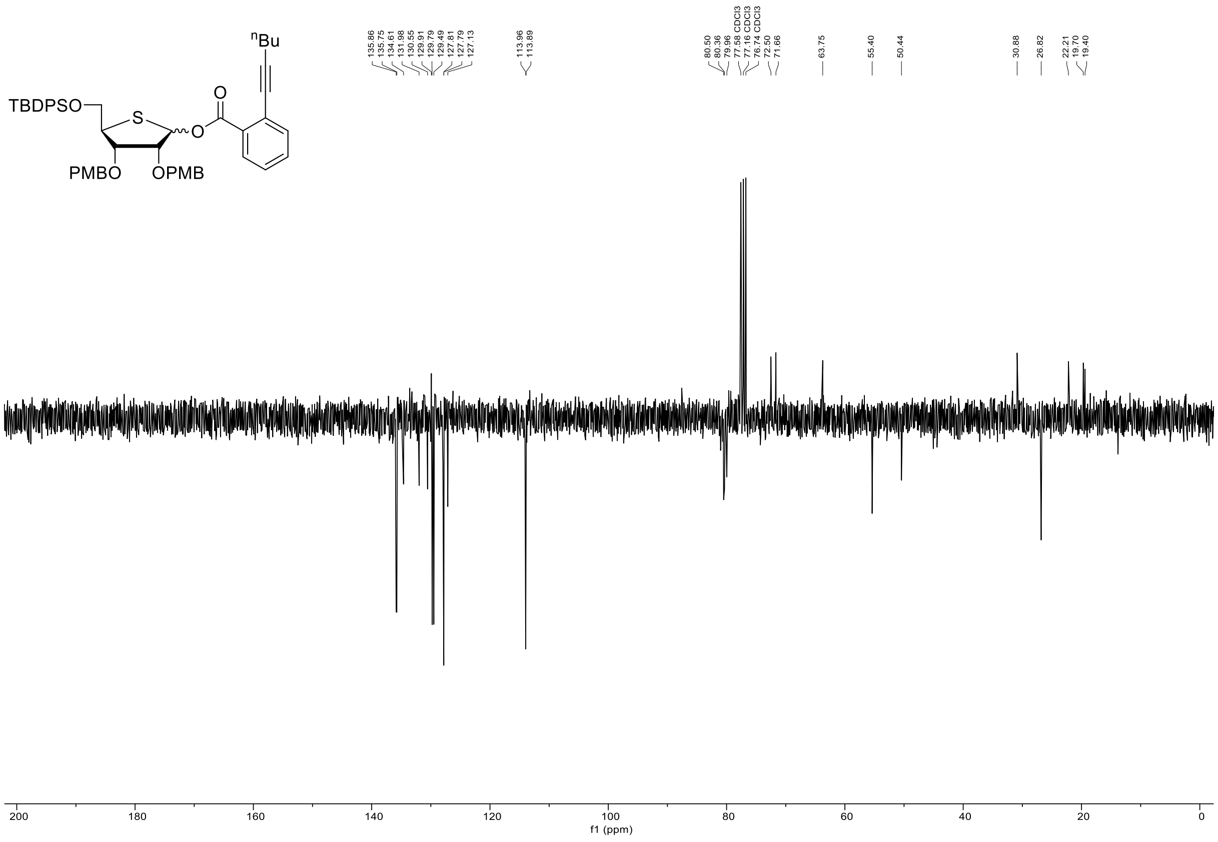

(E)-prop-1-en-1-yl N-(((9H-fluoren-9-yl)methoxy)carbonyl)-O-((2S,3R,4S,5R)-5-(((tert-butyldiphenylsilyl)oxy)methyl)-3,4-bis((4-methoxybenzyl)oxy)tetrahydrothiophen-2-yl)-L-serinate (13)

^{1}H-NMR, CDCl3, 400 MHz

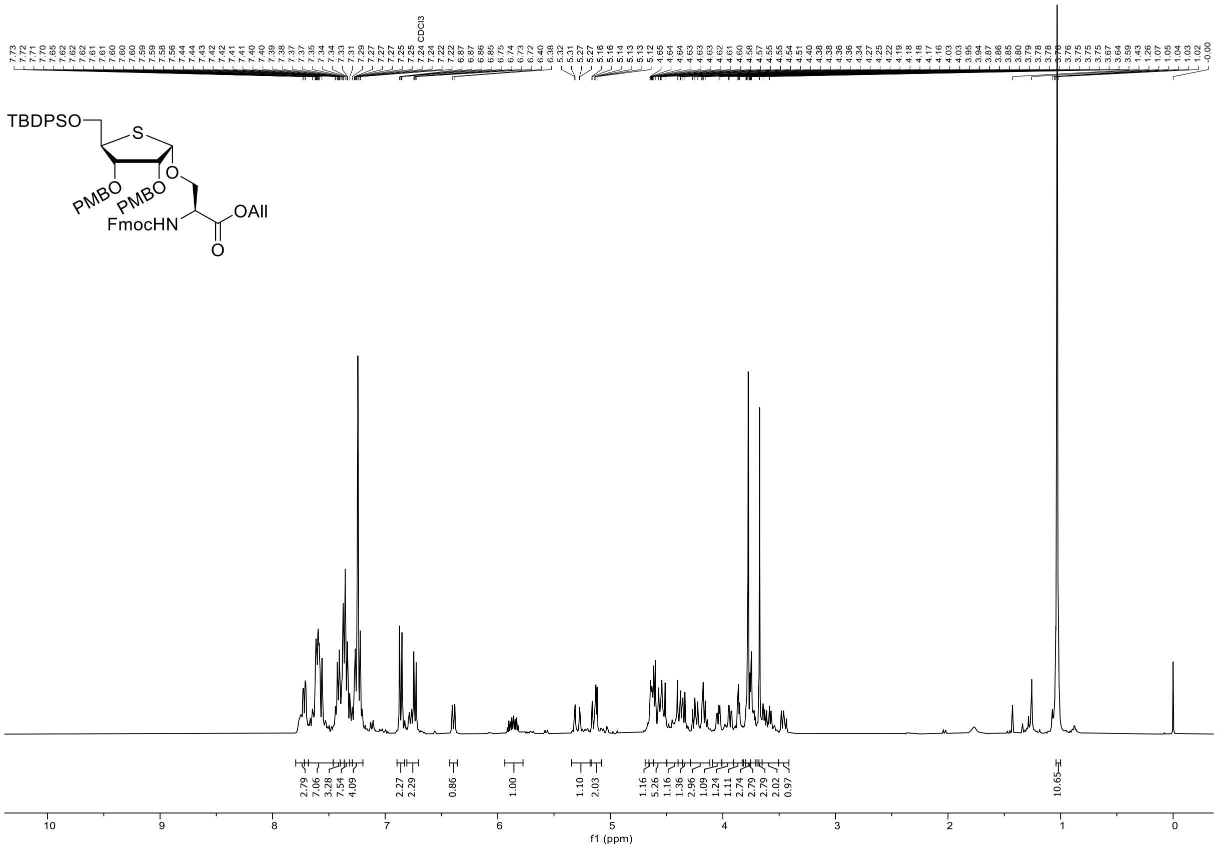

^{13}C-NMR, CDCl3, 101 MHz

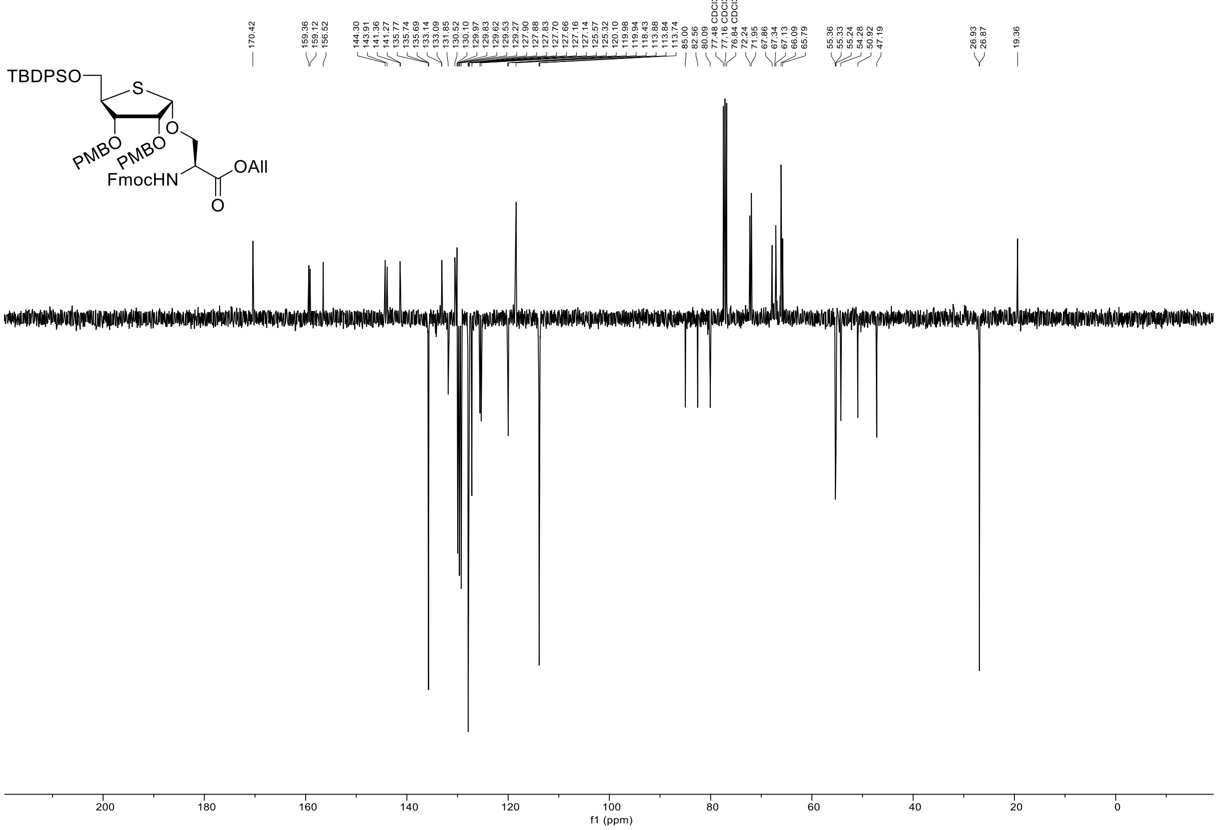

N-(((9H-fluoren-9-yl)methoxy)carbonyl)-O-((2S,3R,4S,5R)-5-(((tert-butyldiphenylsilyl)oxy)methyl)-3,4-bis((4-methoxybenzyl)oxy)tetrahydrothiophen-2-yl)-L-serine (1)

^{1}H-NMR, CDCl$_3$, 400 MHz

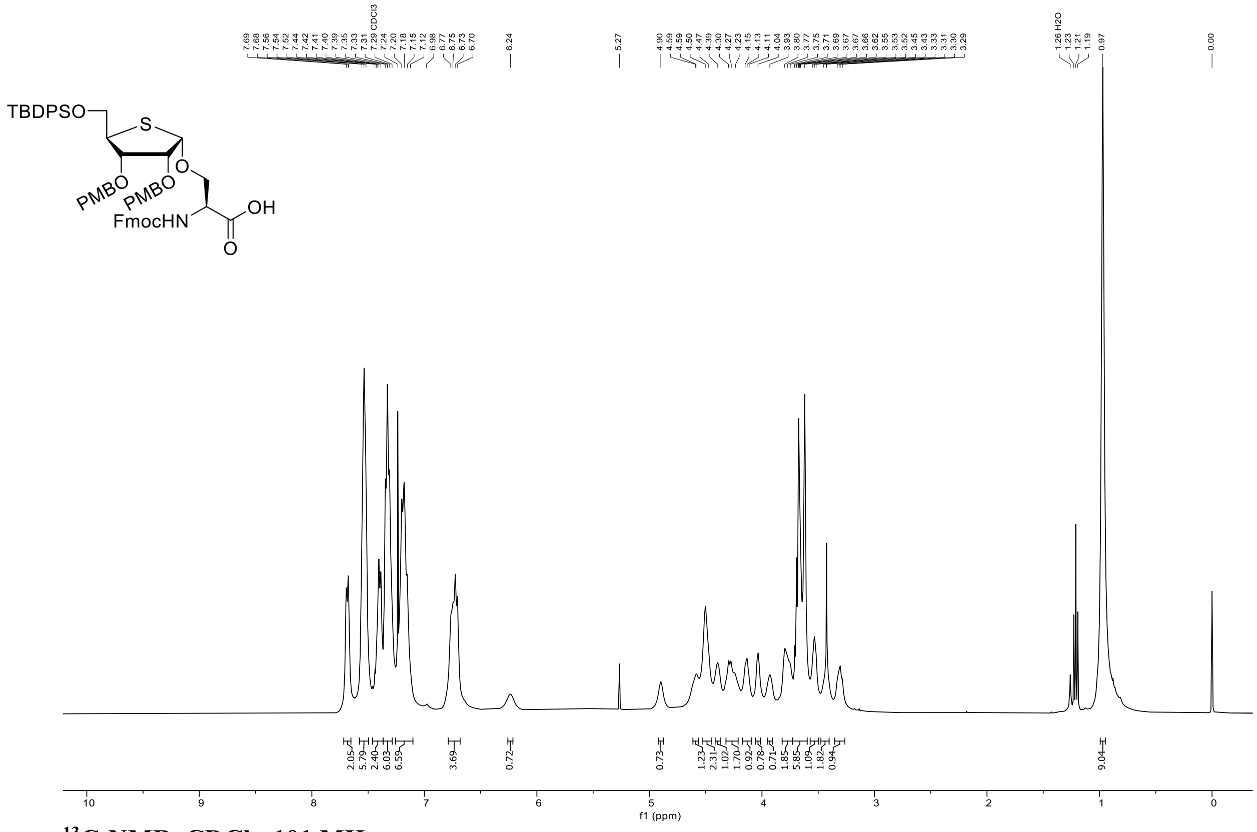

^{13}C-NMR, CDCl$_3$, 101 MHz

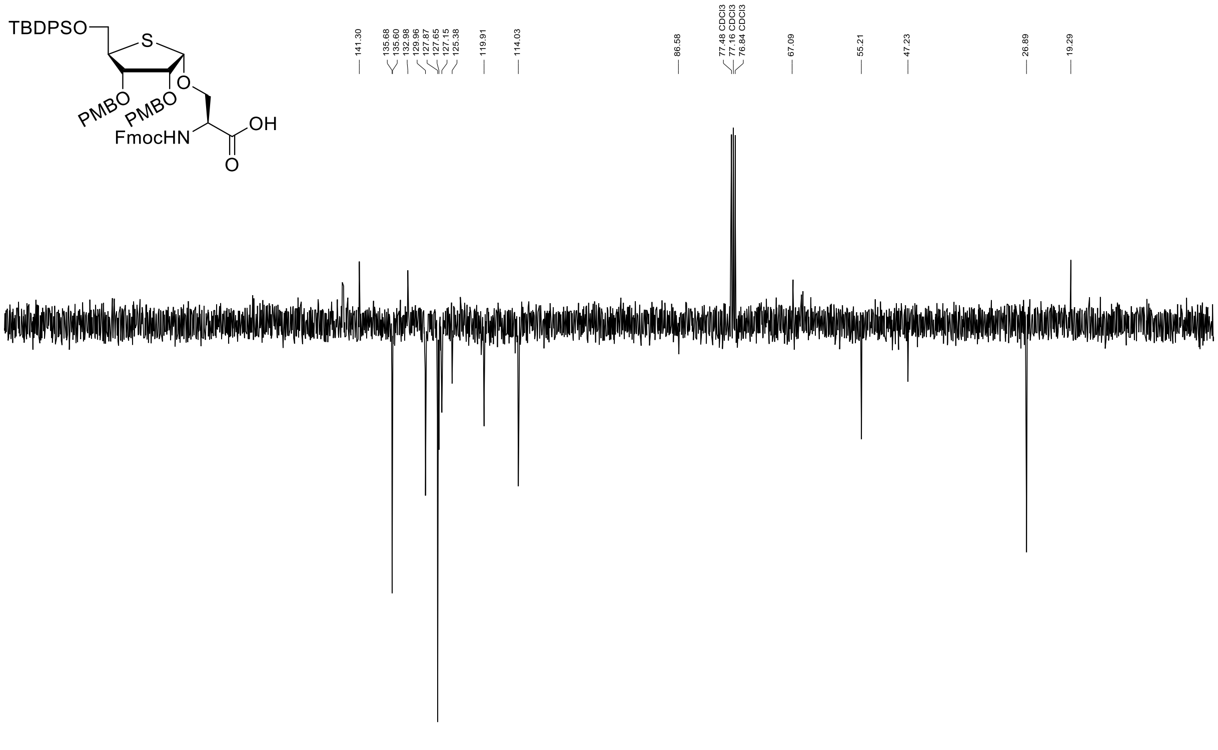

Ac-Pro-Ala-Lys-Ser(5-O-adenosine diphosphate-α-D-4-thio-ribosyl)-Ala-Pro-Ala-Pro-Lys-Lys-Gly-OH (19)

^{1}H-NMR, D_2O, 850 MHz

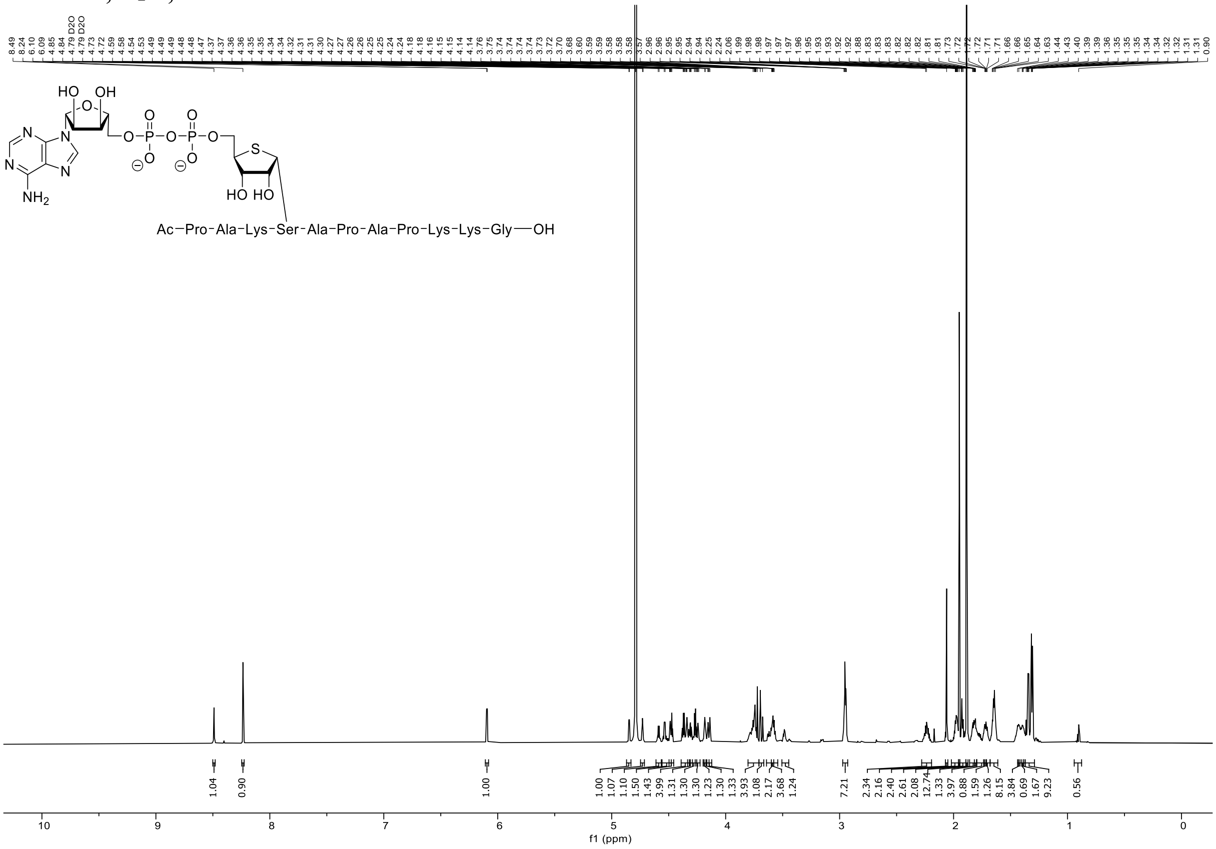

^{31}P-NMR, D_2O, 202 MHz

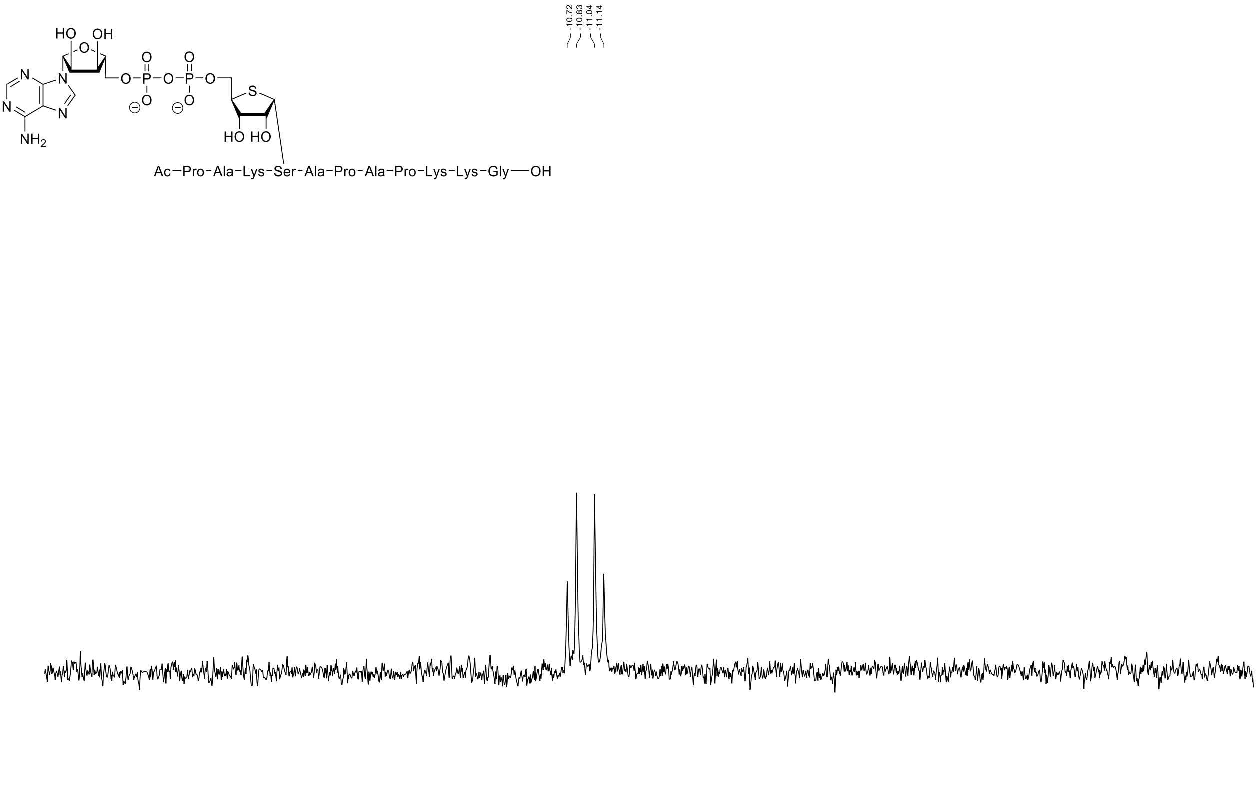

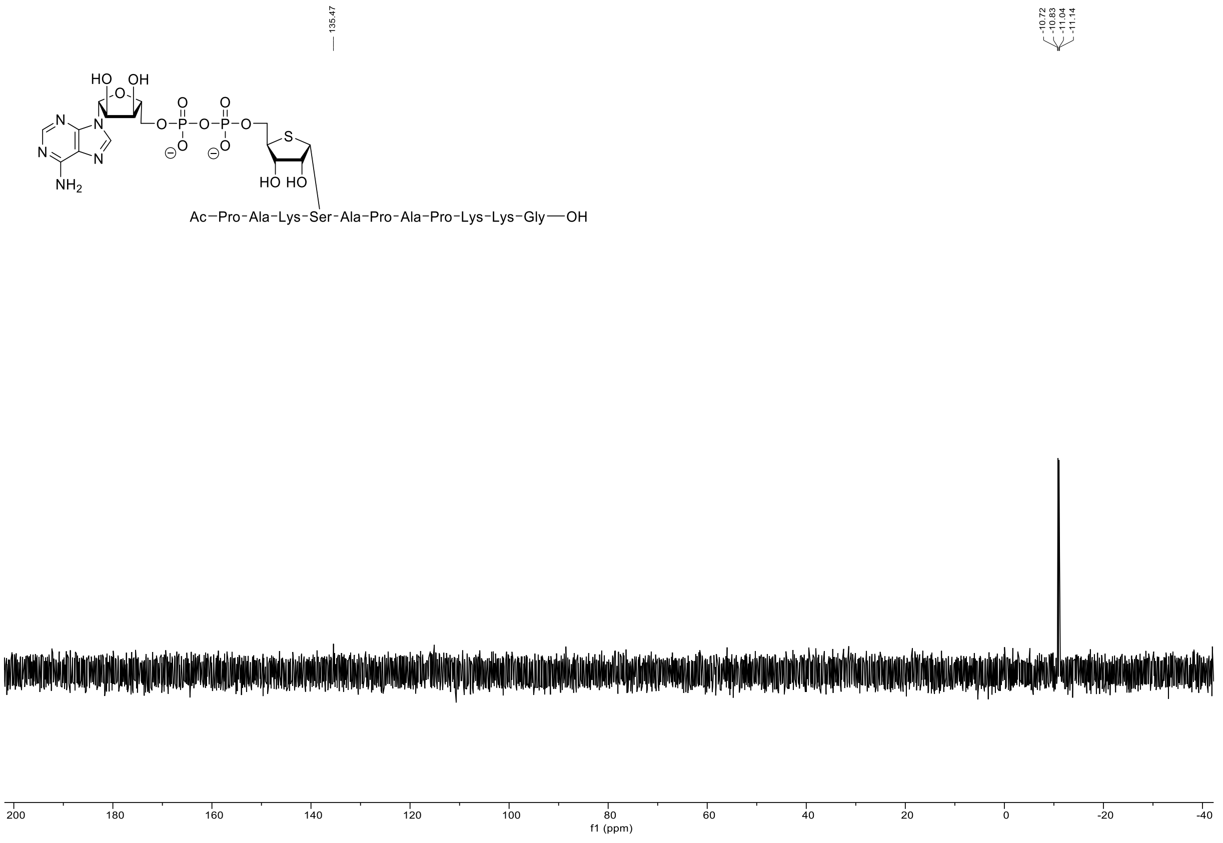

135.47
-10.72
-10.83
-11.04
-11.14
HO
OH
NH2
Ac–Pro–Ala–Lys–Ser–Ala–Pro–Ala–Pro–Lys–Lys–Gly—OH
200
180
160
140
120
100
80
60
40
20
0
-20
-40
f1 (ppm)

LCMS

Ac-Pro-Ala-Lys-Ser(5-O-adenosine diphosphate-α-D-4-thio-ribosyl)-Ala-Pro-Ala-Pro-Lys-Lys-Gly-OH (19)

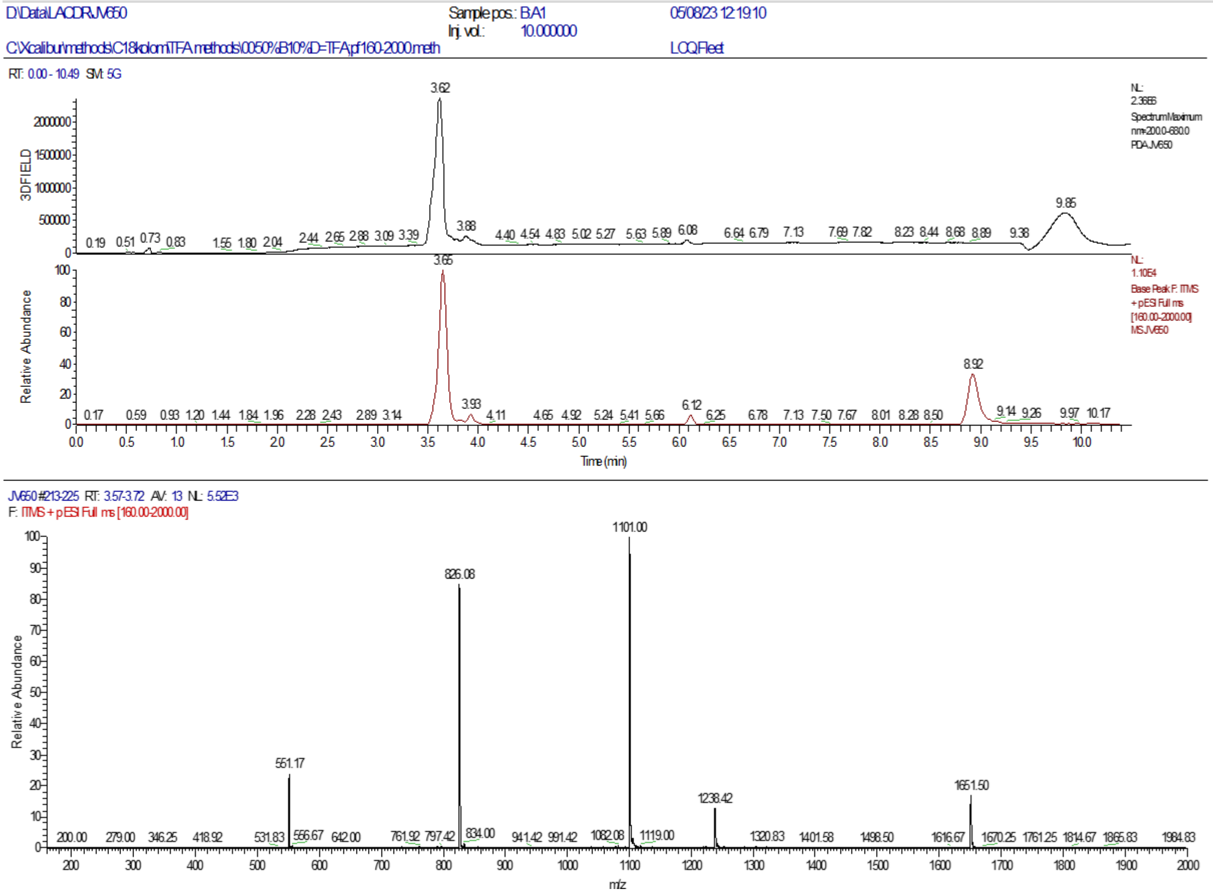

Supplement: Supplementary file 1 — ol3c01554_si_001.pdf [file ol3c01554_si_001.pdf]
